# MTBP phosphorylation controls DNA replication origin firing

**DOI:** 10.1038/s41598-021-83287-w

**Published:** 2021-02-19

**Authors:** Pedro Ferreira, Verena Höfer, Nora Kronshage, Anika Marko, Karl-Uwe Reusswig, Bilal Tetik, Christoph Dießel, Kerstin Köhler, Nikolai Tschernoster, Janine Altmüller, Nina Schulze, Boris Pfander, Dominik Boos

**Affiliations:** 1 Vertebrate DNA Replication Lab, Center of Medical Biotechnology, University of Duisburg-Essen, 45117 Essen, Germany; 2 Max Planck Institute of Biochemistry, DNA Replication and Genome Integrity, 82152 Martinsried, Germany; 3 Imaging Center Campus Essen, Center of Medical Biotechnology, University of Duisburg-Essen, 45117 Essen, Germany; 4 Cologne Center for Genomics (CCG), University of Cologne, Weyertal 115b, 50931 Cologne, Germany; 5 Institute of Human Genetics, University Clinics Cologne, Kerpener Strasse 34, 50931 Cologne, Germany

**Keywords:** Eukaryote, Genetics, Chromosomes, Cell biology, Post-translational modifications, Phosphorylation, DNA replication, Fragile sites, Origin firing

## Abstract

Faithful genome duplication requires regulation of origin firing to determine loci, timing and efficiency of replisome generation. Established kinase targets for eukaryotic origin firing regulation are the Mcm2-7 helicase, Sld3/Treslin/TICRR and Sld2/RecQL4. We report that metazoan Sld7, MTBP (Mdm2 binding protein), is targeted by at least three kinase pathways. MTBP was phosphorylated at CDK consensus sites by cell cycle cyclin-dependent kinases (CDK) and Cdk8/19-cyclin C. Phospho-mimetic MTBP CDK site mutants, but not non-phosphorylatable mutants, promoted origin firing in human cells. MTBP was also phosphorylated at DNA damage checkpoint kinase consensus sites. Phospho-mimetic mutations at these sites inhibited MTBP’s origin firing capability. Whilst expressing a non-phospho MTBP mutant was insufficient to relieve the suppression of origin firing upon DNA damage, the mutant induced a genome-wide increase of origin firing in unperturbed cells. Our work establishes MTBP as a regulation platform of metazoan origin firing.

## Introduction

The eukaryotic genome must be duplicated completely and accurately to guarantee inheritance of stable genomes. Origin firing needs to be efficient and appropriately placed and timed to avoid replication stress and genetic instability. Failure of origin firing regulation can generate replication stress^1,2^, but has been little investigated. In principle, failure of firing regulation can result in low-efficiency firing (under-firing), excessive firing or mis-localised ectopic firing. Global and local under-firing is expected to generate non-replicated gaps by increased inter-origin distances^3–5^. Global under-firing also reduces the overall number of replisomes, which results in increased replication fork speed that was proposed to generate replication stress^6^. Excessive origin firing may deplete cells of back-up origins required to fully replicate the genome in adverse replication conditions and in normal conditions in specific difficult-to-replicate genomic locations^2^. Excessive firing could also result in replication gaps through competition of origins for limiting origin firing factors^7–9^. Ectopic origin firing may generate replication stress by increased collision of replication machines with chromatin processes such as transcription^10,11^.

The main regulation step of origin firing is pre-initiation complex (pre-IC) formation as exemplified by classic cell cycle and DNA damage checkpoint regulations of replication initiation in yeast^2,12^. Pre-IC formation is the first step of converting pre-replicative complexes (pre-RCs), the replicative helicase in a chromatin-loaded inactive form, into active replisomes. In yeast, rising activities of Dbf4-dependent kinase (DDK) and cyclin-dependent kinase (CDK) in S phase mediate the assembly of pre-initiation complexes (pre-ICs) on pre-RCs, comprising Sld3-Sld7 (both form a constitutive complex), Sld2, Dpb11, DNA polymerase epsilon, Cdc45 and GINS^8,13–20^. Sld3-Sld7, Sld2 and Dpb11 dissociate and the active Cdc45-Mcm2-7-GINS-polymerase epsilon replicative helicase and finally mature replisomes form^21–24^. The exact role of Sld7, the yeast orthologue of MTBP^25,26^, has not been elucidated. Sld7 is important but not essential for origin firing in yeast^27^. It binds to Sld3 using its N-terminus and forms Sld7 homo-dimers using its extreme C-terminal domain^28^.

Sld7 is not considered a highly regulated factor, unlike the critical CDK and DDK substrates Sld3, Sld2 and pre-RCs^15,18,19,29^. Moreover, the Rad53 DNA damage kinase phosphorylates both Dbf4 and Sld3 to inhibit the firing of late origins to avoid excessive replication of damaged DNA templates^30–32^.

In addition to the described fundamental regulations by the cell cycle and the DNA damage checkpoint, fine tuning the levels and activities of pre-IC factors is critical for origin firing timing and replication fidelity^7,8,33–35^. For example, the DNA damage kinase Mec1 has basal activity in the absence of exogenous DNA damage^36^, and the ATR-dependent kinase pathway in metazoa was found to attenuate origin firing in the absence of induced DNA damage^37,38^.

**Figure 1:**
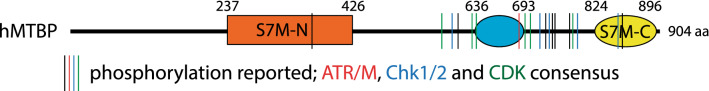
Domain architecture of human MTBP with reported phosphorylation sites. Schematic of the MTBP protein. Reportedly phosphorylated consensus sites for ATR/M (S/T-Q, red, T687), Chk1/2 (R/K-x-x-S/T, blue, T577, S738, S755, T804, S846) and CDK (S/T-P, green, S539, T635, S639, S703, S707, T799) are indicated by vertical lines (see main text for references). S7M-N, -C, Sld7-MTBP amino and carboxy-terminal domains; blue oval, metazoa-specific domain; aa, amino acids; numbers, aa positions in human MTBP.

In metazoa, the detailed molecular processes of pre-IC formation are unknown. All core yeast pre-IC factors have orthologues in metazoa^25,26,39–44^. Also conserved is the essential role of Treslin/TICRR and TopBP1, metazoan Sld3 and Dpb11, in mediating CDK-dependent S phase-specific origin firing^45,46^. Like Sld3, Treslin/TICRR appears to be a target to fine-regulate origin firing^47–49^. MTBP is essential for replication in human cells and *Xenopus* egg extracts^26,50^. The N-terminal Sld7-homologous domain (S7M-N, for Sld7-MTBP N-terminal domain) (Fig. 1) facilitates replication through binding to Treslin/TICRR in human cells^25^. Its C-terminal Sld7-homologous domain (S7M-C, Sld7-MTBP C-terminal domain) may mediate origin firing through homo-dimerization^25,28^. The metazoa-specific MTBP middle domain has multiple roles in replication, one of which involves interaction with the Cdk8/19-cyclin C kinase that is required to prevent under-replication by unknown mechanisms^25^.

MTBP was originally identified using yeast-two-hybrid experiments as a binder of the Mdm2 protein that helps Mdm2 degrade p53^51,52^. Since then MTBP has been implicated in mitosis^53^, cell migration^54^, transcription^55^ and cancer formation^56,57^. The relevance of these findings for MTBP’s role in replication remains unexplored.

We here put MTBP into the spotlight as an origin firing regulation platform specifically in metazoa. It is targeted by at least three kinase pathways, Cdk8/19-cyclin C^25^, cell cycle CDK and by phosphorylation at DNA damage kinase consensus sites. Eliminating MTBP regulation through phosphorylation changed origin firing frequency in normal cell growth conditions. Our insight highlights that understanding how metazoa replicate their vast genomes accurately and completely requires considering metazoa-specific origin firing regulations in addition to those widely conserved.

## Results

### MTBP is posttranslationally modified

Searching online databases revealed that MTBP is modified by phosphorylation, ubiquitylation, SUMOylation and methylation (phosphosite.org). Modifications are particularly numerous in the C-terminal half of MTBP containing the metazoa-specific central and the S7M-C regions (Fig. 1). Initial experiments showed that mutations eliminating a methylation site (lysine 739) or eight lysines reported to be ubiquitylated (positions 570, 591, 604, 608, 627, 630, 642, 752)^58–62^ or SUMOylated (752) had no effect on the capability of MTBP to support incorporation of the nucleotide analogue BrdU in Hela cells (Supplementary Information Fig. S1), showing that these modifications are not essential for overall DNA synthesis.

**Figure 2:**
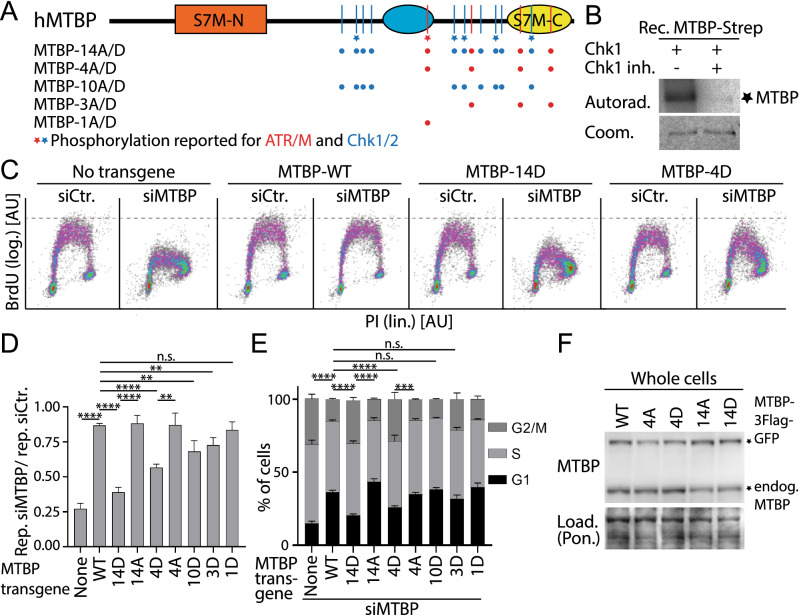
Phosphorylation of MTBP at checkpoint kinase consensus sites inhibits genome replication. (**A**) Domain architecture of human MTBP with mutated consensus phosphorylation sites for ATR/M (S/T-Q, red) (amino acids T687, S761, S827, S858) and Chk1/2 (R/K-x-x-S/T, blue) (amino acids T531, T577, S579, T611, S738, S755, T781, T804, S808, S846). *, reported phosphorylations (phospho-site.org). Mutations to aspartate (D) or alanine (A) introduced in MTBP are indicated by colour-coded dots: MTBP-14A/D, 14 ATR/M and Chk1/2 mutated; 4/3/1A/D, all four, three or one ATR/M site(s) mutated; 10A/D, Chk1/2 sites mutated. (**B**) Chk1 in vitro phosphorylation of MTBP. Recombinant 6His-Treslin/TICRR-1-1258-MTBP-Strep was incubated with Chk1 in the presence of γ‐^32^P‐ATP and DMSO or Chk1 inhibitor AZD7762 and detected by autoradiography. Coomassie staining controlled loading (Load.); Rec., recombinant. (**C**) Flow cytometry density plots of HeLa Flip-In T-Rex cells expressing siMTBP-resistant C-terminally 3xFlag-TEV2-GFP-tagged MTBP-wild type (WT), MTBP-14D, MTBP-4D or no transgene, treated with control siRNA (siCtr) or siRNA against MTBP (siMTBP) and doxycycline, stained with anti-5 bromo-2′deoxyuridine (BrdU) after pulse-labelling and with propidium iodide (PI). Log./lin., logarithmic/linear scale; [AU], arbitrary units. MTBP-14D; MTBP-4D mutants, see A) (**D**,**E**) Quantification of replication activity (**D**) or cell cycle distribution (**E**) using BrdU-PI-flow cytometry as described in C. Error bars, SEM. n = 11 (no transgene); 9 (MTBP-WT); 8 (MTBP-14D); 3 (MTBP-14A); 8 (MTBP-4D); 5 (MTBP-4A); 3 (MTBP-10D); 4 (MTBP-3D); 3 (MTBP-1D); MTBP mutants: see A; significance tests: parametric, unpaired, two tailed student t-test. Significance tests in E) indicate differences in G2/M population distribution. (**F**) Whole cell lysates of stable cell lines described in C were analysed by immunoblotting using anti-MTBP (12H7), and Ponceau (Pon.) staining.

We realised that MTBP is phosphorylated at consensus sites for CDK and DNA damage checkpoint kinases. All six CDK consensus sites (pS/T-P) (Fig. 1) in MTBP were reported to be phosphorylated^63–66^. Out of the 23 checkpoint kinase consensus sites (four ATM/R sites (S/T-Q) and 19 Chk1/2 sites (R/KxxS/T)) in MTBP six sites in the heavily modified C-terminus were found phosphorylated (Fig. 2A). One of them is a consensus site for the ATR/M kinases and five for Chk1/2^58,67,68^. We decided to investigate the role of CDK and checkpoint kinase sites further, because these pathways are known regulators of origin firing.

### MTBP phosphorylation at checkpoint kinase consensus sites inhibits genome replication

Recombinant Chk1 phosphorylated recombinant MTBP, as determined by incorporation of radioactive phosphate from γ-^32^P-ATP into recombinant MTBP (Fig. 2B). We did not find evidence for DNA damage induction of the reported MTBP phosphorylation in 293T and Hela cells using phospho-peptide mass spectrometry and phospho-specific antibodies. This suggests that this phosphorylation is DNA damage independent or that it is effectively removed by cellular phosphatases.

To test if phosphorylation of MTBP at checkpoint kinase consensus sites regulates origin firing we generated non-phosphorylatable and phospho-mimetic mutants by exchange to alanine (A) or aspartate (D), respectively. Because mutating all 23 sites (MTBP-23A/D) lowered expression levels (Supplementary Information Fig. S2), perhaps due to destabilising protein folding, we focused on mutants with 14 exchanges (MTBP-14A/D) in the heavily modified middle and C-terminal MTBP regions (Fig. 2A). MTBP-14A/D expressed to the same levels as MTBP-WT (Supplementary Information Fig. S2). To assess the replication-inducing capacity of these mutants, we used RNAi to replace endogenous MTBP with siRNA-resistant mutants using isogenic Hela cell FlipIn cell lines, as described^25,50^ (Supplementary Information Fig. S3). Overall DNA synthesis was measured by flow cytometry after pulse-labelling cells with 5-Bromo-2′-deoxyuridine (BrdU) in combination with staining double stranded DNA by propidium iodide (PI). Cells expressing siRNA-resistant wild type MTBP (MTBP-WT) transgene showed normal BrdU-PI profiles regardless of treatment with control (siCtr) or MTBP siRNA (siMTBP) (Fig. 2C–E). In contrast, cells expressing MTBP-14D and no-transgene control cells had strongly reduced replication signals upon siMTBP treatment to 39% and 27%, respectively, compared to 87% with MTBP-WT (Fig. 2C,D). MTBP-14D and control cells also had increased G2/M populations with a concomitant dramatic drop of the G1 population (Fig. 2E). Flow cytometry-determined G2/M populations often increase upon compromising replication as a consequence of delays in G2 and M phases. The defects with MTBP-14D reflected an effect of phosphorylation at these sites rather than unspecific MTBP inactivation due to the mutations, because MTBP-14A behaved like MTBP-WT (Fig. 2D,E). Expression levels of MTBP-14D, 14A and WT were similar (Fig. 2F). We then separated the mutations of MTBP-14A/D. Mutating the four ATR/M sites to D (MTBP-4D), but not to A (4A) (Fig. 2A), partly suppressed replication (Fig. 2C–F). Mutating three ATR sites (3D) and mutating all ten Chk1 sites to D (10D) (Fig. 2A) had moderate consequences if any (Fig. 2D,E Supplementary Information Fig. S4).

We conclude that phosphorylation at ATR/M and Chk1/2 consensus sites of MTBP cooperate to inhibit replication.

### Origin firing is inhibited by MTBP phosphorylation at checkpoint kinase consensus sites

**Figure 3:**
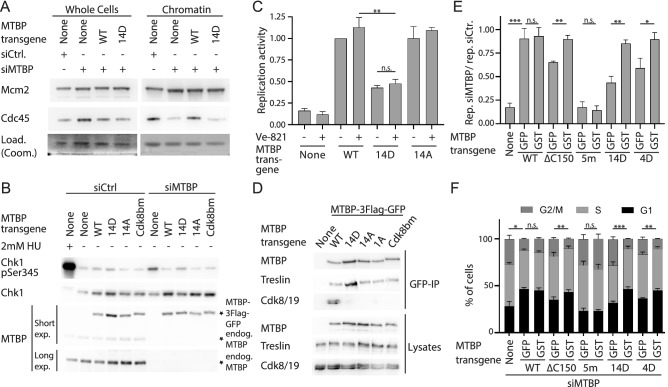
MTBP inhibition by phosphorylation blocks origin firing, involving suppression of the S7M-C homo-dimerization domain. (**A**) Whole cell lysates and chromatin fractions isolated from Hela Flip-In T-Rex cells expressing siMTBP resistant C-terminally 3xFlag-TEV2-GFP-tagged MTBP-wild type (WT), MTBP-14D or no transgene after siMTBP or siCtr treatment were immunoblotted using antibodies against MTBP (12H7), Mcm2 and Cdc45. Coomassie staining controlled loading (Load.). (**B**) Whole cell lysates of the indicated siCtr or siMTBP-treated Hela-FlpIn cell lines were immunoblotted using anti-MTBP (12H7), anti-Chk1, anti-pS345-Chk1. Treatment with hydroxurea (HU) was used to control for ATR signalling. (**C**) Replication activity as measured by BrdU-PI flow cytometry of the indicated cell lines upon siMTBP treatment as described in Fig. 2D. n = 3, error bars: SEM, significance tests as in 2D/E. (**D**) C-terminally 3xFlag-TEV2-GFP-tagged MTBP-wild type (WT), MTBP-14D, MTBP-14A, MTBP-1A or MTBP-Cdk8bm were transiently transfected into 293T cells before analysis by anti-GFP immunoprecipitation (IP) and immunoblotting using antibodies against MTBP (12H7), Treslin/TICRR (117) and Cdk8; MTBP mutants: MTBP-1A, amino acid exchange T687A; MTBP-Cdk8bm, amino acid exchanges L620D, P622A, L623D, F632A, V633D, L634D, T635A. (**E**,**F**) Quantification of replication (**E**) or cell cycle distribution (**F**) in the indicated Hela Flip-In T-Rex cell lines as described in 2D/E. n = 4 (no transgene); 4 (MTBP-WT); 4 (MTBP-ΔC150); 4 (MTBP-5 m), 3 (MTBP-14D), 3 (MTBP-4D-GS4-GFP); 5 (MTBP-4D-GS4-GST); MTBP mutants: MTBP-ΔC150, C-terminal 150 amino acids deleted; MTBP-5 m, amino acid exchanges V306D, I309D, D313A, L314D, P315D.

We next tested if MTBP phosphorylation at the checkpoint kinase sites inhibits replication by suppressing origin firing. To test if specifically the origin firing step is inhibited in MTBP-14D expressing cells, we isolated chromatin from siRNA-treated control cells or cells after RNAi-replacement of endogenous MTBP with MTBP-WT or 14D. Anti-Mcm2 immunoblotting served as a marker for pre-RCs and anti-Cdc45 for replisomes (origin firing). Control cells confirmed that MTBP siRNA specifically suppressed origin firing but not pre-RC formation^50^ (Fig. 3A). siMTBP-treated MTBP-WT and 14D expressing cells showed similar levels of pre-RCs, but MTBP-14D had Cdc45 levels that were as low as with siMTBP-treated control cells, indicating that phosphorylation at the 14 checkpoint kinase sites suppresses origin firing.

### MTBP-14D is rescued by fusing a homodimerization domain, like deletion mutants of the Sld7-homologous C-terminal domain

We next asked how MTBP phosphorylation inhibits MTBP’s replication activity. We first tested if the inhibition of replication in MTBP-14D cells is an indirect consequence of the MTBP-14D protein activating the ATR-Chk1 DNA damage checkpoint that is known to inhibit origin firing. Western blotting against the ATR substrate site phospho-S345-Chk1 showed moderately (compared to HU treatment) but reproducibly increased ATR signalling in cells expressing MTBP-14D (Fig. 3B, Supplementary Information Fig. S5) (the non-CDK binding mutant MTBP-Cdk8bm^25^ was used here as a control because MTBP-14D/A do not strongly bind Cdk8/19-cyclin C (see below)). However, the checkpoint signal was not specific to MTBP-14D expression, because no-transgene control cells showed similarly increased phospho-S345-Chk1 signals upon siMTBP treatment, suggesting that the checkpoint is activated as a consequence of severely compromised DNA replication. We then checked if ATR inhibition by the chemical compound VE-821 relieves the block of replication in MTBP-14D cells. Although VE-821 mildly increased replication in unperturbed cells, indicating basal checkpoint activity even in normal growth conditions, VE-821 was not particularly effective in restoring replication in MTBP-14D expressing cells (Fig. 3C, Supplementary Information Fig. S5). In contrast, VE-821 effectively relieved the block of origin firing upon ionising radiation (discussed below). Thus, ATR signalling is not required for replication inhibition by MTBP-14D, which is consistent with the model that checkpoint site phosphorylation of MTBP directly inhibits replication origin firing.

We then tested which molecular activity of MTBP is inhibited in MTBP-14D. Figure 3D shows that MTBP-14D is proficient in the essential binding to Treslin/TICRR. Binding to Cdk8/19-cyclin C was compromised in both MTBP-14A and 14D (Fig. 3D). The interaction with Cdk8/19-cyclin C is required to complete DNA replication but has little effect on overall BrdU incorporation^25^, indicating that inability to interact with the kinase is insufficient to explain the inactivity of MTBP-14D.

We then tested if phosphorylation inhibits the MTBP-S7M-C domain. We reported earlier that the S7M-C domain supports replication probably by mediating MTBP homodimerization based on the finding that MTBP mutants that lack S7M-C (MTBP-∆C150 and ∆C80), which show partly reduced replication inducing activity, were rescued by fusing the dimerising GST but not the non-dimerising GFP^25^ (Fig. 3E,F). MTBP-14D and 4D, but not the Treslin/TICRR non-binding MTBP-5m, were rescued by GST, whereas fusing GFP had no effect (Fig. 3E,F, Supplementary Information Fig. S6). The observation that specifically the phospho-mimetic MTBP mutants show the same rescue behaviour by GST fusion as mutants of the S7M-C homodimerization domain suggested that the phosphorylation may inhibit MTBP by counteracting the activity of the S7M-C domain. The inhibitory effect of 14D on replication was stronger than that of deleting S7M-C. Phospho-mimicry must therefore inhibit additional activities required for normal replication. These inhibited activities do not include the binding to Treslin/TICRR (Fig. 3D). As a side note, the rescue of MTBP-14D by GST fusion provides additional evidence that the 14 mutations do not inactivate MTBP by misfolding.

### MTBP phosphorylation at the 14 checkpoint kinase consensus sites is dispensable for origin firing suppression upon DNA damage, but required for normal origin firing rates in unperturbed cells

**Figure 4:**
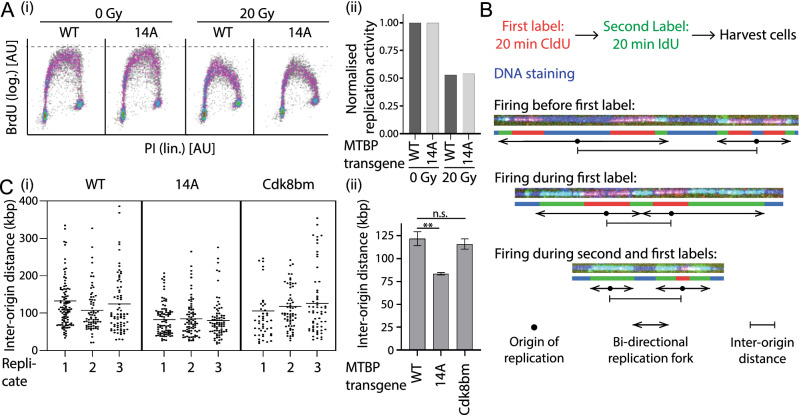
Phosphorylation of MTBP at checkpoint kinase consensus sites inhibits origin firing in unperturbed cell growth conditions. (**A**) Flow cytometry PI-BrdU density plots of HeLa Flip-In T-Rex cells expressing siMTBP resistant C-terminally 3xFlag-TEV2-GFP-tagged MTBP-wild type (WT) or MTBP-14A treated with siMTBP, doxycycline, irradiated with 0 Gy or 20 Gy, and stained as in 2C. (i), density plots and (ii) quantification of replication signals, involving normalisation to cells irradiated with 0 Gy. Log., logarithmic scale; lin., linear scale; [AU], arbitrary units. Significance tests as in 2D/E. (**B**,**C**) Inter-origin distance measured by DNA combing of siMTBP-treated Hela Flip-In T-Rex cells expressing siMTBP resistant C-terminally 3xFlag-TEV2-GFP-tagged MTBP-wild type (WT), MTBP-14A or MTBP-cdk8bm. C(i), scatter plots of individual experiments; C(ii), quantifications of respective means; kbp, kilobasepairs. Error bars: SEM; n = 3; significance tests as in 2D/E. See also Supplementary Information Fig. S10.

MTBP phosphorylation at checkpoint kinase sites could help inhibit origin firing upon DNA damage. Failure to inhibit firing results in radio-resistant DNA synthesis (RDS). If bypassing MTBP inhibition was sufficient to relieve the block of origin firing upon DNA damage expressing MTBP-14A should induce RDS. Control experiments showed that BrdU-PI flow cytometry of cells treated with ionising radiation (IR) was able to monitor ATR-dependent (VE-821-sensitive) origin firing inhibition (Supplementary Information Fig. S7). Expressing MTBP-14A did not result in RDS (Fig. 4A). Thus, bypassing MTBP phosphorylation did not relieve origin firing inhibition upon DNA damage. This indicates that other origin firing inhibition pathways are sufficient to prevent RDS, but does not exclude a contribution of MTBP phosphorylation.

We asked next if MTBP phosphorylation at checkpoint kinase consensus sites has a DNA damage-independent origin firing regulation role. We used DNA combing to test if expression of MTBP-14A increases origin firing frequency in cells growing in the absence of exogenous DNA damage. MTBP-14A cells showed a reduction of average inter-origin distance (IOD) by 32% from 122 kbp of MTBP-WT expressing cells to 83 kbp in the mutant, indicating elevated firing (Fig. 4B,C). The IOD reduction was not due to the inability of MTBP-14A to bind Cdk8/19-cyclin C because the MTBP-Cdk8bm mutant, which cannot bind Cdk8/19-cyclin C due to seven amino acid exchanges in the metazoa-specific middle domain^25^, showed a normal IOD (Fig. 4C). The reduced IOD with MTBP-14A also did not result from a change of the DNA damage checkpoint signalling status, which could involve dormant origin firing, as determined by anti-phospho-S345-Chk1 western blotting (Fig. 3B, Supplementary Information Fig. S8), and also not from decreased passive replication of origins due to slower forks, because average fork speed was normal in MTBP-14A cells (Supplementary Information Fig. S8). In summary, the finding that MTBP-14 cells fire more origins is consistent with the model that the 14 alanine mutations bypass an attenuation mechanism of origin firing in normal growth conditions.

As described, excessive origin firing could result in replication stress. However, the observations that neither elevated basic levels of phospho-S345-Chk1 in normally growing MTBP-14A cells was detected (Fig. 3B) nor an elevated response to titrating hydroxyurea (Supplementary Information Fig. S8) argued against significant replication stress inMTBP-14A cells.

### Phosphorylation of MTBP at its six CDK consensus sites impacts replication origin firing

**Figure 5:**
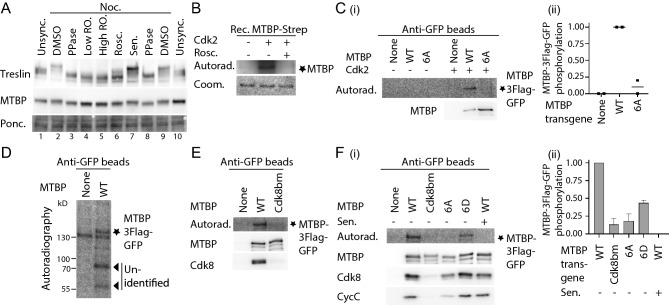
MTBP is phosphorylated by cell cycle CDK and Cdk8/19-cyclin C. (**A**) Phosphorylation-mediated gel shifts of MTBP and Treslin/TICRR in mitotic cells depend on M-CDK. Cells arrested in mitosis with nocodazole (Noc) or asynchronous cells were treated for 30 min with DMSO, low (9 µM) or high concentrations (90 µM) of RO-3306 (RO.), roscovitine (Rosc.) or senexin A (Sen.). After cell lysis lysates were treated with lambda phosphatase (PPase) where indicated before immunoblotting using antibodies against MTBP (12H7) and Treslin/TICRR (148). (**B**) In vitro phosphorylation of MTBP by S-CDK. Recombinant 6His-Treslin/TICRR-aa1-1258-MTBP-Strep was incubated with Cdk2-cyclin A in the presence of γ‐^32^P‐ATP and DMSO or CDK inhibitor roscovitine and detected by autoradiography. Coomassie staining controlled loading (Load.). Rec., recombinant. (**C**) S-CDK phosphorylation of MTBP depends on CDK consensus sites. C-terminally 3xFlag-TEV2-GFP-tagged MTBP-wild type (WT), MTBP-6A or control plasmid were transiently transfected into 293T cells before anti-GFP IP, incubation with buffer or Cdk2-Cyclin A in the presence of γ‐^32^P‐ATP, and detection by autoradiography and immunoblotting using an antibody against MTBP (12H7). (i), autoradiogram; (ii), quantification of signal intensities of two independent replicates. WT signals were assigned the value 1 and control plasmid 0. MTBP-6A, amino acid exchanges S539A, T635A, S639A, S703A, S707A, T799A. (**D**,**E**) MTBP phosphorylation depends on binding to Cdk8/19-cyclin C. Native lysates of cells transfected as in C were immunoprecipitated and incubation with γ‐^32^P‐ATP before detection by autoradiography or immunoblotting using an antibody against MTBP (12H7) and Cdk8. (**F**) Native lysates of cells transfected with the indicated MTBP versions and with Cdk8-cyclin C were analysis as in D in the presence of DMSO or Cdk8 inhibitor senexin A. (i), autoradiogram; (ii), quantification of signal intensities of three independent replicates. WT signal intensities were assigned the value 1 and Cdk8 inhibitor treatment 0.

Previous evidence suggested that MTBP is phosphorylated in lysates from mitotic cells, as judged by a gel shift upon nocodazole arrest in comparison with unsynchronised cells and cells from G1 phase^25,50^. We found that MTBP and Treslin/TICRR, which is a heavily phosphorylated cell cycle CDK substrate^45,50^, migrated faster in lysates of nocodazole-arrested cells upon treatment of the lysate with lambda phosphatase (Fig. 5A, lanes 3,8; Supplementary Information Fig. S9). This showed that the reported gel shift was indeed due to phosphorylation. Treatment with low concentrations of the CDK inhibitor RO-3306 (lane 4) effectively reversed the MTBP and Treslin/TICRR gel shifts (Supplementary Information Fig. S9). Low RO-3306 concentrations effectively inhibit Cdk1, which constitutes M-phase CDK kinases (M-CDK), but not Cdk2-dependent kinases. Also the Cdk1/2 inhibitor roscovitine (lane 6), but not the Cdk8/19 inhibiting senexin A (lane 7), partly reversed the gel shift (Supplementary Information Fig. S9). Less pronounced phosphatase-sensitive gel shifts of MTBP and Treslin/TICRR were also detectable in cells synchronised in S phase by a 4 h release from a nocodazole-thymidine arrest (Supplementary Information Fig. S9). However, kinase inhibitor-treated samples were inconclusive for MTBP due to the lower degree of gel shift in S phase cells. These experiments suggested that M-CDK phosphorylates MTBP, and that MTBP is also phosphorylated in S phase, although the contributions of S-CDK and Cdk8/19-cyclin C remain unclear.

To test if S-CDK can phosphorylate MTBP at the six CDK consensus sites in the protein we used recombinant Cdk2-cyclin A for in vitro kinase experiments. Figure 5B shows that recombinant MTBP indeed incorporated radioactive phosphate upon incubation with recombinant Cdk2-cyclin A. The kinase did not phosphorylate GFP-3Flag-tagged MTBP isolated from lysates of transfected 293T cells when all six CDK consensus sites were mutated to alanine (MTBP-6A) (Fig. 5C(i),(ii)), showing that the phosphorylation depends on the six CDK consensus sites. Together, these experiments suggested that MTBP is a substrate of M and S-CDK kinases. M-CDK phosphorylates MTBP in mitotic cells, whereas the contribution of S-CDK to MTBP phosphorylation in S phase cells remains open.

We found evidence that MTBP can also be phosphorylated by Cdk8-19-cyclin C. Incubating beads that contained MTBP-3Flag-GFP immunoprecipitated from cell lysates with γ-^32^P-ATP we noticed that a kinase activity co-purified that produced a band at the size of MTBP-3Flag-GFP (Fig. 5D). This kinase activity was only detectable in long exposures, which is why it was not evident in similar kinase experiments involving Cdk2-cyclin A (Fig. 5B). The co-purifying activity depended on the binding of MTBP to Cdk8/19-cyclin C since the signal was undetectable with MTBP-Cdk8bm that cannot bind to Cdk8/19-cyclin C (Fig. 5E). To improve signal intensity, we co-transfected Cdk8-cyclin C together with MTBP mutants for the following experiments. MTBP phosphorylation was eliminated by the Cdk8/19-cyclin C inhibitor senexin A (Fig. 5F). Also the CDK site mutant MTBP-6A nearly abrogated the signals (Fig. 5F). Because MTBP-6A binds Cdk8/19-cyclin C less well than MTBP-WT (Fig. 5F(i)) we tested a phospho-mimetic aspartate mutant of MTBP (MTBP-6D) that binds Cdk8/19-cyclin C normally. Quantification of three experiments showed that MTBP-6D incorporated ^32^P detectably, but 2.3 times less than MTBP-WT (Fig. 5F(ii)). These experiments suggested that Cdk8/19-cyclin C phosphorylates MTBP on the six CDK consensus sites and also on non-CDK consensus sites. Whether Cdk8/19-cyclin C also phosphorylates MTBP in cells remains open, because senexin A treatment had only little if any effect on the MTBP phospho-shift in M phase cells (Fig. 5A; Supplementary Information Fig. S9).

**Figure 6:**
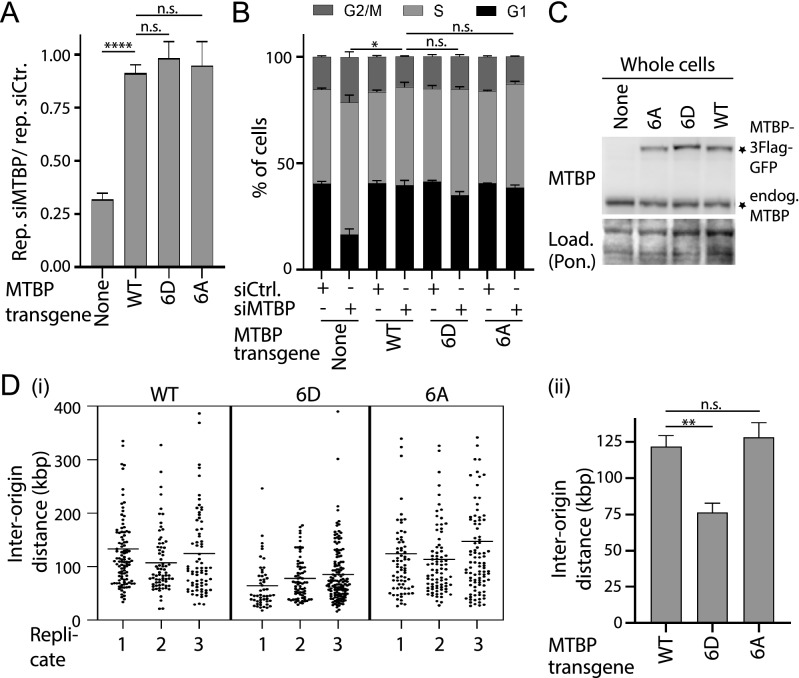
Phosphorylation of MTBP on CDK consensus sites promotes origin firing in unperturbed growth conditions. (**A**,**B**) Quantification of replication activity (**A**) or cell cycle distribution (**B**) using BrdU-flow cytometry as described in 2D/E of indicated HeLa Flip-In T-Rex cell lines. Error bars, SEM. n = 3; MTBP mutants: MTBP-6A/D, see 5C. Significance tests as in 2D/E. (**C**) Whole cell lysates of stable cell lines described in A were analysed by immunoblotting using anti-MTBP (12H7), and Ponceau (Pon.) staining. (**D**) Inter-origin distance of cells as described in A measured by DNA combing as described in 4C. See also Supplementary Information Fig. S10.

We then investigated the relevance of MTBP phosphorylation at CDK consensus sites on the replication function of MTBP by analysing the phospho-mimetic and non-phosphorylatable MTBP-6D and the MTBP-6A mutants. Neither mutant had overt effects on BrdU incorporation, cell cycle distribution and expression levels, indicating that MTBP phosphorylation by CDK is not essential for replication (Fig. 6A–C). DNA combing showed that MTBP-6D reduced the IOD by 38% from 122 kbp (MTBP-WT) to 76 kbp (Fig. 6D). MTBP-6A did not affect IOD. We then tested whether the increased origin firing (decreased IOD) in MTBP-6D cells could be an indirect consequence of replication stress-induced dormant origin firing. However, we found no evidence for elevated checkpoint signalling in MTBP-6D cells, as basic phospho-S345-Chk1 levels and the response to increasing hydroxyurea concentrations were indistinguishable from MTBP-WT cells (Supplementary Information Fig. S8). Together, these experiments with MTBP CDK site mutants indicated that more origins fired in cells where phosphorylation was mimicked. This phenotype could reflect a direct or indirect effect of CDK site phosphorylation on origin firing frequency. Average replication fork speed was normal in the MTBP-6D mutant (Supplementary Information Fig. S8).

We conclude from Figs. 1–6 that MTBP phosphorylation by several kinase pathways controls origin firing in normally growing cells.

## Discussion

Understanding the cellular and molecular mechanisms that guarantee that origin firing occurs with sufficient efficiency at the right time in the right place is a central biological question as it is key to inheriting stable genomes. Understanding the consequences of failure of this firing control is important both biologically and from the perspective of human health, because DDK inhibitors that inhibit firing are considered as cancer therapeutics^69,70^.

### MTBP is a regulation focus of origin firing

Our insight of MTBP as a focus of kinase regulation places MTBP among the more established origin firing regulators in metazoa. Like yeast Sld7, MTBP is a core factor of pre-IC formation^25,50^. Pre-IC formation is well-placed for origin firing regulation, as it is the most upstream essential firing step^14,17,20^. In yeast and metazoa, other pre-IC proteins, namely Sld3/Treslin/TICRR, Sld2/RecQ4, the Mcm2-7 helicase and Dbf4, are known to mediate fundamental cell cycle and DNA damage checkpoint-mediated origin firing regulation and fine tuning of origin firing, involving a variety of molecular mechanisms that control levels and activities of these pre-IC factors^7,8,15,18,19,30,34,35,44–49,71–74^.

Our GST-fusion experiments (Fig. 3E,F) suggested that MTBP regulation may occur by counteracting the S7M-C domain that probably mediates MTBP homo-dimerization^25,28^. MTBP dimerization may help mediate bi-directionality of replication by ensuring that both Mcm2-7 hexamers of pre-RCs, but never only one, are converted into replisomes. S7M-C has also been suggested to have DNA binding activity^26^.

### MTBP phosphorylation may contribute to establishing origin firing homeostasis

What are the cellular mechanisms of regulating genome replication by MTBP phosphorylation at checkpoint kinase and CDK consensus sites?

The genome-wide attenuation of origin firing by MTBP phosphorylation at checkpoint kinase consensus sites supports the concept that origin firing regulation guarantees firing homeostasis. Origin firing homeostasis depends on the right propensity of the genomic origins to fire in order to avoid replication stress by excessive firing or by under-firing. We could not identify which kinase executes phosphorylation at the 14 checkpoint kinase consensus sites in cells. However, previous observations that DNA damage checkpoint kinases control the efficiency of origin firing in unperturbed conditions^37,38^ indicated that the increased firing in cells expressing the MTBP-14A mutant may result from bypassing MTBP inhibition by a basal global activity of DNA damage checkpoint kinases in the absence of exogenous DNA damage.

As an origin firing homeostasis mechanism MTBP phosphorylation could go hand in hand with other mechanisms that control overall levels of origin firing factors and their firing promoting activity. For example, pre-IC proteins were shown to be limiting for origin firing in vertebrates and yeasts and specific control mechanisms exist to determine their levels^7,9,34,35^. Moreover, fast recycling dynamics of such limiting pre-IC factors from fired origins could help guarantee that sufficient pre-IC factors are available for firing origins downstream. Yeast Sld2, Sld3 and Dpb11 are presumably present upon origin firing in a CDK phosphorylation-dependent complex^17–19^ and may require active disassembly and extraction from the activated Mcm2-7 helicase to fire the next origin.

Origin firing homeostasis may also depend on the balance between firing-regulating kinases and phosphatases. Rif1 in association with protein phosphatase 1 were proposed to inhibit origin firing by counteracting DDK phosphorylation^75–79^. Specific kinase-phosphatase balance mechanisms may also exist for other firing-regulating kinases. Rising S-CDK activity levels towards late S phase could tip the respective balance to fire late origins with low intrinsic firing propensity.

Intuitively, an imbalance of origin firing homeostasis by a decrease in firing propensity should result in less efficient firing and replication stress through large IODs that result in non-replicated gaps. Our observation that depletion of MTBP and replacement of endogenous MTBP by MTBP-14D induce DNA damage signalling support this idea, confirming similar earlier findings^6^ (Fig. 3B and Supplementary Information Figs. S5, S8). Interestingly, firing moderately more origins than normal by expressing MTBP-14A and MTBP-6D did not result in detectable replication stress in Hela cells, suggesting that a slight imbalance of origin firing homeostasis by increased origin firing propensity is not sufficient not cause major problems to unperturbed cells.

### Potential role of MTBP phosphorylation by Cdk8/19-cyclin C and cell cycle CDK in origin firing regulation

Our observation that MTBP-6D cells showed increased origin firing is consistent with this phosphorylation promoting origin firing directly or indirectly. Because MTBP-6A cells showed no decrease in overall firing and BrdU incorporation, a role of this phosphorylation in Hela cells, perhaps a role in controlling a specific subset of origins, remains unproven.

We reported earlier that preventing Cdk8/19-cyclin C binding to MTBP increased replication stress signs, whilst affecting BrdU incorporation mildly^25^. We found here that the six CDK consensus sites in MTBP (or a subset of them) can be phosphorylated by Cdk8/19-cyclin C co-purified with MTBP, but that this kinase also phosphorylates non-CDK consensus sites in this assay, which remain to be mapped. Perhaps, the recruitment of Cdk8/19-cyclin C by tight binding to MTBP enables the kinase to phosphorylate non-consensus sites. Given that Cdk8/19-cyclin C is a transcriptional CDK it is tempting to speculate that this kinase phosphorylates MTBP to coordinate transcription with replication to avoid genetic alterations^11^.

Cell cycle CDK phosphorylation of MTBP occurred in mitotic cells. Thus, cell cycle CDK phosphorylation of MTBP may serve mitotic roles^53^ apart from potential roles in origin firing.

Our establishing of MTBP as a platform of origin firing regulation shows that understanding how metazoa facilitate origin firing regulation to faithfully inherit their genetic information will require investigating MTBP along-side the previously established firing regulators.

## Materials and methods

For all immunoblots blots, radiograms and other protein gels presented in main and supplemental figures, uncropped images are shown in Supplementary Fig. S11.

### Cell culture

Cell culture conditions and generation of stable HeLa Flip-In T-Rex cell lines (kind gift from G Muller-Newen^80^) using pcDNA5 derivates were described^25^.

### Transient transfection of 293T cells

Transient 293T (ATCC CRL-11268) cell transfections were conducted as described using CaCl_2_^25^ or using PEI (polyethylenimine) according to a protocol kindly shared by David Cortez’ lab. 4 µg plasmid DNA in 100 µl DMEM without serum were combined with 24 µl PEI (1 mg/ml) and incubated for 15 min before addition to 5 × 10^6^ cells on a 10 cm dish. Transfected cells were used 72 h post transfection. For subsequent immunoblotting of whole cell lysates harvested cells were boiled in Laemmli loading buffer.

### RNAi

RNAi using RNAiMax (Life Technologies) was described^25^. The following siRNAs were used: siCtr/GL2, CGUACGCGGAAUACUUCGAUU (CTM-134302, Dharmacon)*; siMTBP: 1:1 mix of* MTBP1,GAGAGAAACAGUUAGCUAA/MTBP2, UCACAUUGUUGGAUGCUAA (J-013953-06-0050 and J-013953-08-0050, Dharmacon)*;* Treslin/TICRRGAACAAAGGTTATCACAAA (custom-made, Dharmacon).

### Antibodies

Antibodies against Treslin/TICRR, MTBP, Cdk8-cyclin C, GFP and Flag, as well as HRP-coupled antibodies were described^25^.

anti-Mcm2 (goat, Santa Cruz (sc-9839)); Chk1 (mouse, Santa Cruz (sc-8404); Chk1 pSer345 (rabbit, Cell Signalling (2348S)).

### Immunoprecipitation

Co-immunoprecipitation experiments were done as described previously with the indicated amendments^25^. In brief, C-terminally 3FLAG-TEV2-GFP tagged MTBP was immunoprecipitated from lysates of a 10 cm tissue culture plate each of transiently transfected 293T cells after lysis in 5 times the pellet volume of lysis buffer (20 mM HEPES, 300 mM NaCl, 5 mM EDTA, 10% Glycerol, complete EDTA-free protease inhibitor cocktail (Roche, 05056489001), 0,1% Triton X100, 2 mM 2-mercaptoethanol). Lysates were incubated with 20 µg anti-GFP nanobodies covalently coupled to 10 µl NHS sepharose (GE Healthcare, 10343240). After incubation for 2 h at 4 °C the immunoprecipitates were washed with lysis buffer and retained proteins eluted by boiling in Laemmli sample buffer.

For immunoprecipitations of C-terminally 3FLAG-TEV2-GFP tagged MTBP from 293T cells for in vitro phosphorylation of MTBP a 15 cm plate of transiently transfected 293T cells were lysed in 5 × pellet volume of lysis buffer (replacing EDTA with EGTA). After incubation for 2 h at 4 °C the immunoprecipitates were used for in vitro kinase assays.

### Chromatin preparation

Chromatin-enriched fractions were purified from HeLa Flip-In T-Rex cell lines as described^25^.

### Purification of 6His-Treslin/TICRR-aa1-1258-MTBP-strep complex

Two liters of Sf9 insect cells were co-infected with the individual viruses for pLib-6xHis-Treslin/TICRR-1-1258 and pLib-MTBP-Strep. 72 h after infection, cells were lysed in 120 ml of lysis buffer (20 mM HEPES pH 8.0, 500 mM NaCl, 0.1% Tween20, 25 mM Imidazole, 0.5 mM TCEP, protease inhibitor cocktail (Roche Complete protease inhibitor cocktail, 05056489001)). The lysate was centrifuged at 44,800×*g* for 30 min and the supernatant was incubated with Strep-Tactin agarose beads for 1 h at 4 °C. After three washes of the beads with washing buffer (20 mM HEPES pH 8.0, 500 mM NaCl, 0.01% Tween20, 25 mM Imidazole), elution was done with wash buffer supplemented with 2.5 mM desthiobiotin. The eluates were purified over a 1 ml HiTrap HP Ni–NTA column (GE Healthcare), using a linear gradient of from 25 mM to 1 M imidazole in elution buffer (20 mM HEPES pH8.0, 500 mM NaCl, 0.01% Tween20). Peak fractions were pooled.

### In vitro phosphorylation of MTBP

For in vitro kinase assays, 500 ng recombinant human 6His-Treslin/TICRR-aa1-1258-MTBP-strep or anti-GFP sepharose-bound MTBP-Flag-GFP immunoprecipitated from transiently transfected 293T cells were incubated at 30 °C for 30 min with kinase buffer (20 mM Hepes pH 8,0, 300 mM NaCl, 13,5 mM MgCl2, 10 mM B-Glycerol-phosphate, 5 mM B-Merc, 20 mM EGTA), 1 µl of recombinant Cdk2-Cyclin A (2 mg/ml)^45^ or 1 µl of recombinant Chk1 (generous gift from S Jackson) and, where indicated, with DMSO, 200 µM of CDK inhibitor roscovitine or 200 nM of Chk1 inhibitor AZD7762, or 30 µM senexin A in the presence of γ‐^32^P‐ATP and kinase buffer. For Cdk8/19-cyclin C kinase assays, no recombinant kinase was added. Detection required long exposures for autoradiography or co-transfection of Cdk8 and cyclin C. Laemmli SDS sample buffer was added for SDS PAGE analysis and autoradiography. Quantification of signal intensities and background subtraction were done using Fiji/ImageJ software.

### Cell cycle flow cytometry

Pulse-labelling of cells for 15–30 min with 10 µM 5-bromo-2′-deoxyuridine (BrdU) and subsequent detection of BrdU with anti-BrdU-FITC and double stranded DNA with propidium iodide were described^25^. Flow cytometry analysis was performed using a FACSCalibur (BD Bioscience) or a MACSQuant (Miltenyi Biotec) flow cytometers.

Data analysis was performed using the Kaluza Analysis 1.3 software (Beckman Coulter). Cell aggregates were excluded based on their high FL2-W signal. BrdU-PI profiles were generated as density plots. For quantifications of replication activities, the BrdU signal of BrdU-positive S phase cells was background-subtracted using the signal intensity of BrdU-negative G1 and G2/M phase cells to calculate the replication-dependent BrdU signal. For quantifications of the efficiency of replication rescue by MTBP transgenes, the BrdU-replication signal from MTBP/Treslin/TICRR siRNAi treat cells was divided by the BrdU-replication signal of control siRNA treated cells. Visualisation as bar diagrams and statistics was done using GraphPad Prism 5 or 8 (GraphPad Software Inc.).

### Radio resistant DNA synthesis (RDS)

HeLa Flip-In T-Rex cells were treated with DMSO or 6 µM ATR inhibitor VE821 for 1 h before being gamma irradiated at 130 kV with a 0.5 mm-Al-filter at a dose rate of 1 Gy/72 s (RX-650 Faxitron) at room temperature. Control cells not irradiated were left outside at room temperature for the duration of the gamma irradiation procedure. Cells were then grown at standard conditions for 1 h (or indicated times) and BrdU-labelled for cell cycle flow cytometry.

### DNA combing

Cells were labelled for 20 min each with 20 µM 5-Chloro-2′-deoxyuridine (CldU) and subsequently with 200 µM 5-Iodo-2′-deoxyuridine (IdU). Cells were washed in cold PBS before harvesting with trypsin. Cells were embedded in agarose (8 × 10^5^ cells in 90 µl) before treatment with Proteinase K (Sigma Aldrich, 3115844001) over night at 42 °C followed by washing the agarose plugs in TE 10.1 buffer (10 mM Tris/HCl, pH8, 1 mM EDTA pH8) first three times for 1 h then once for 2 h. Plugs were transferred into 1.5 ml 0,5 M MES and melted at 68 °C for 40 min followed by 20 min at 42 °C before adding 5 µl β-Agarase (NEB, M0393L) to each sample and incubation overnight. DNA was combed on salinized coverslips (Genomic Vision, COV-002-RUO) using the FiberComb machine (Genomic Vision). Combed coverslips were incubated at 70 °C for 2 h. For immunodetection, DNA was denatured with 0,5 M NaOH/1 M NaCl solution for 8 min at RT. After 3 washing steps with 1XDPBS (LifeTechnologies, 14190169) the coverslips were dehydrated using successive 1 min washes with 70%, 90% and 100% ethanol. After drying the coverslips were incubated with 25 µl Block Aid solution (Invitrogen, B10710) on a glass slide. Antibody incubation was performed as followed, indicated amounts were used per coverslip: 1st antibody (4 µl mouse anti-BrdU (anti-BrdU, clone B44, BD Bioscience, 347580) + 2 µl rat anti-BrdU (Bio-Rad, OBT0030G) in 19 µl Block Aid) for 1 h at 37 °C, 2nd antibody (2 µl goat anti-mouse Cy3 (Abcam, AB6946) + 2 µl goat anti-rat Cy5 (Abcam, AB6565) in 21 µl Block Aid) for 45 min at 37 °C, 3rd antibody (4 µl mouse anti-ssDNA (http://dshb.biology.uiowa.edu/autoimmune-ssDNA) in 21 µl Block Aid) for 2 h at 37 °C, 4th antibody (2 µl goat anti-mouse BV480 (Jackson Immuno Research, 115-685-166) in 23 µl Block Aid) for 45 min at 37 °C. Between antibody incubations coverslips were washed 3 times with 1xPBS, 0.05% Tween for 3 min at 100 rpm on an orbital shaker.

Coverslips were then dehydrated by successive incubations in 70%, 90% and 100% ethanol. Image acquisition was performed using the FiberVision scanner (Genomic Vision). Each coverslip was mounted onto a barcoded coverslip carrier (Genomic Vision S.A., 92220 Bagneux. France) and imaged using the Fiber Vision fluorescent microscope (Genomic Vision). Fluorescence imaging was performed automatically using the “RCA standard” program. Data analysis was performed using the web-based FiberStudio software (Genomic Vision, Version 2.0.2). The automated imaging creates merges of the fluorescent channels that were used for subsequent analysis. For fork speed analysis more than 100 structures were validated after visual confirmation of the automated assignment of the FiberStudio Software. For IOD at least 50 inter-origin-distances were measured by only validation of intact structures that were flanked by intact DNA on both sides.

The structures were categorized in origin of replication, termination of replication or ongoing forks. For IOD, mean distance between two adjacent origins in intact fibers was calculated. For fork speed, mean length of intact labeled forks was measured and divided by labeling time. 2 or more replicates were done and the average of the means were calculated for each condition.

## Supplementary Information

Supplementary Information.

## Data Availability

The authors will comply with Nature Research policies for the sharing of research materials and data.
