# Supplementary material for: MTBP phosphorylation controls DNA replication origin firing
Source: Sci Rep. 2021 Feb 19;11:4242. doi: 10.1038/s41598-021-83287-w (PMC7895959; doi:10.1038/s41598-021-83287-w)

Supplementary information

MTBP phosphorylation controls DNA replication origin firing

Pedro Ferreira[1^], Verena Höfer[1^], Nora Kronshage[1], Anika Marko[1], Karl-Uwe Reusswig[2], Bilal Tetik[1], Christoph Dießel[1], Kerstin Köhler[1], Nikolai Tschernoster[4,5], Janine Altmüller[4], Nina Schulze[3], Boris Pfander[2], Dominik Boos[1*]

[1] Vertebrate DNA Replication Lab, Center of Medical Biotechnology, University of Duisburg-Essen, 45117 Essen, Germany

[2] Max Planck Institute of Biochemistry, DNA Replication and Genome Integrity, 82152 Martinsried, Germany

[3] Imaging Center Campus Essen, Center of Medical Biotechnology, University of Duisburg-Essen, 45117, Germany

[4] Cologne Center for Genomics (CCG), University of Cologne, Weyertal 115b, 50931 Cologne, Germany

[5] University Clinics Cologne, Institute of Human Genetics, Kerpener Strasse 34, 50931 Cologne, Germany

[^] equal contribution

[*] corresponding author: dominik.boos@uni-due.de; phone: +49 201 183 4132, ORCID: https://orcid.org/0000-0003-0018-4375

Fig. S1: MTBP mutants of methylation (mut-Met.) and ubiquitylation/sumoylation (met-Ubi.) sites do not result in obvious defects in genome replication.

A/B) Hela Flip-In T-Rex cell expressing RNAi-resistant MTBP-wild type (WT), MTBP mut-Met., MTBP mut-Ubi., or no transgene were treated with control siRNA (siCtr) or siRNA against MTBP (siMTBP) and doxycycline, stained with anti-5 bromo-2'deoxyuridine (BrdU) after pulse-labelling and by propidium iodide (PI). Quantification of replication activity for each cell line is shown in A and cell cycle distribution in B. Error bar: SEM; n =3; MTBP mutants: MTBP-mut-Met., amino acid exchanges, K739A; MTBP-mut-Ubi., amino acid exchanges, K570R, K591R, K604R, K608R, K627R, K630R, K642R, K752R; Significance tests as in 2D/E. In (B) are indicated differences in G2/M population distribution.

C) Expression levels of methylation and ubiquitylation MTBP mutants is similar to MTBP-WT. Whole cell lysates of stable cell lines cells described in C were analysed by immunoblotting using anti-MTBP (12H7) and Ponceau (Pon.) staining.

Figure S1

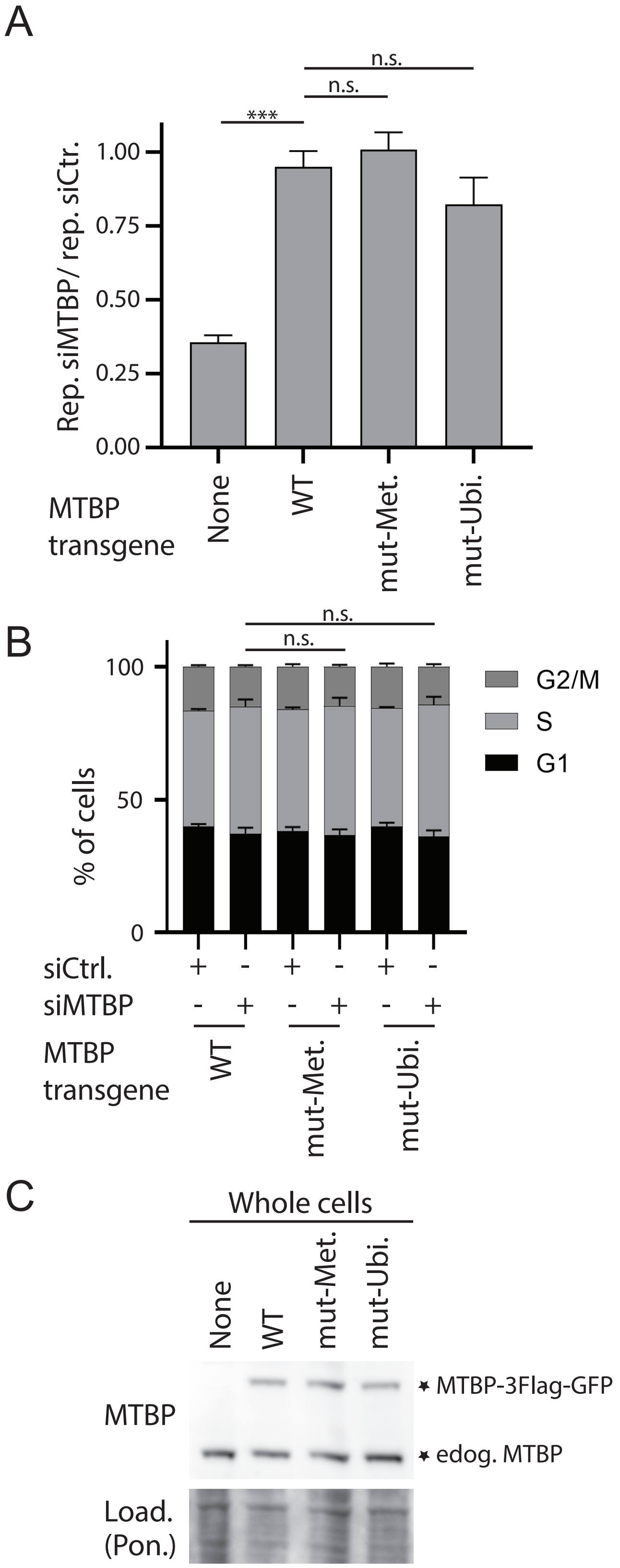
A
n.s.
n.s.
***
Rep. siMTBP/ rep. siCtr.
1.00
0.75
0.50
0.25
0.00
MTBP
transgene
None
WT
mut-Met.
mut-Ubi.
B
n.s.
n.s.
100
50
0
% of cells
G2/M
S
G1
siCtrl.
siMTBP
MTBP
transgene
WT
mut-Met.
mut-Ubi.
C
Whole cells
None
WT
mut-Met.
mut-Ubi.
MTBP
MTBP-3Flag-GFP
edog. MTBP
Load.
(Pon.)

Fig. S2: Expression level of MTBP-23A is lower than MTBP-WT. Soluble cell lysates of 293T cells transiently transfect with C-terminally 3xFlag-TEV2-GFP-tagged MTBP-wild type (WT), MTBP-23A or MTBP-14A were analysed by immunoblotting using anti-MTBP (12H7) and Ponceau (Pon.) staining. MTBP-23A, amino acid exchanges S17A, S55A, T265A, S270A, S336A, T386A, S388A, T439A, T441A, T531A, T577A, S579A, T611A, T687A, S738A, S755A, S761A, T781A, T804A, S808A, S827A, S846A, S858A;

Figure S2

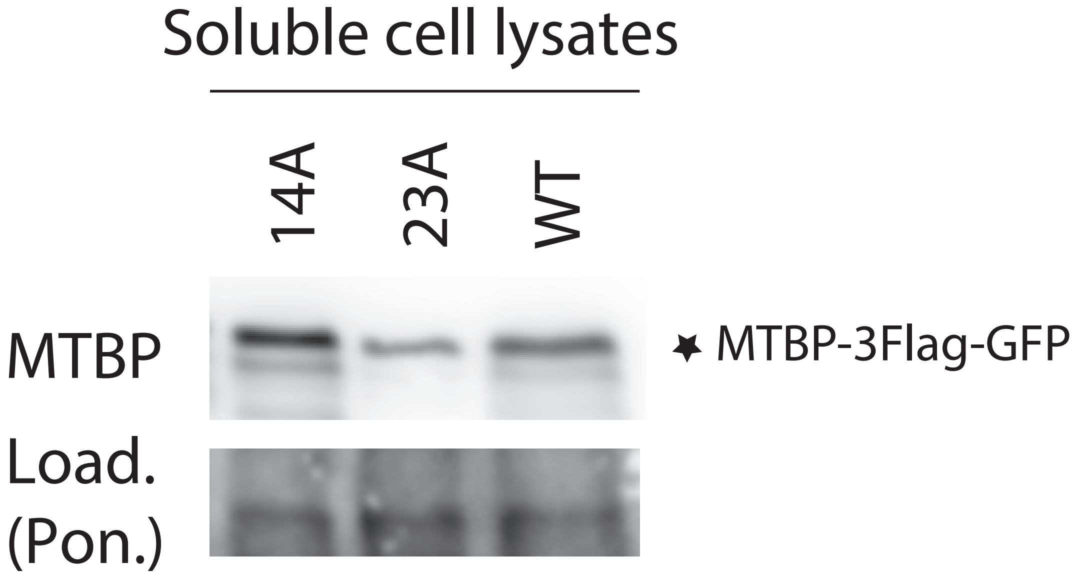

Fig. S3: RNAi-replacement system of endogenous MTBP in Hela-Flip-In cell lines. siRNA against MTBP (siMTBP) specifically eliminates endogenous, but not siRNA-resistant transgenes MTBP. Replacement of endogenous MTBP by siRNA as published [1,2]. Whole cell lysates of Hela Flip-In T-Rex cell expressing RNAi-resistant MTBP-wild type (WT) were treated with control siRNA (siCtr) or siRNA against MTBP (siMTBP) and doxycycline as indicated and analysed by immunoblotting using anti-MTBP (12H7) and Ponceau (Pon.) staining.

1 Boos, D., Yekezare, M. & Diffley, J. F. Identification of a heteromeric complex that promotes DNA replication origin firing in human cells. *Science* **340**, 981-984, doi:10.1126/science.1237448 (2013).

2 Kohler, K. *et al.* The Cdk8/19-cyclin C transcription regulator functions in genome replication through metazoan Sld7. *PLoS Biol* **17**, e2006767, doi:10.1371/journal.pbio.2006767 (2019).

Figure S3

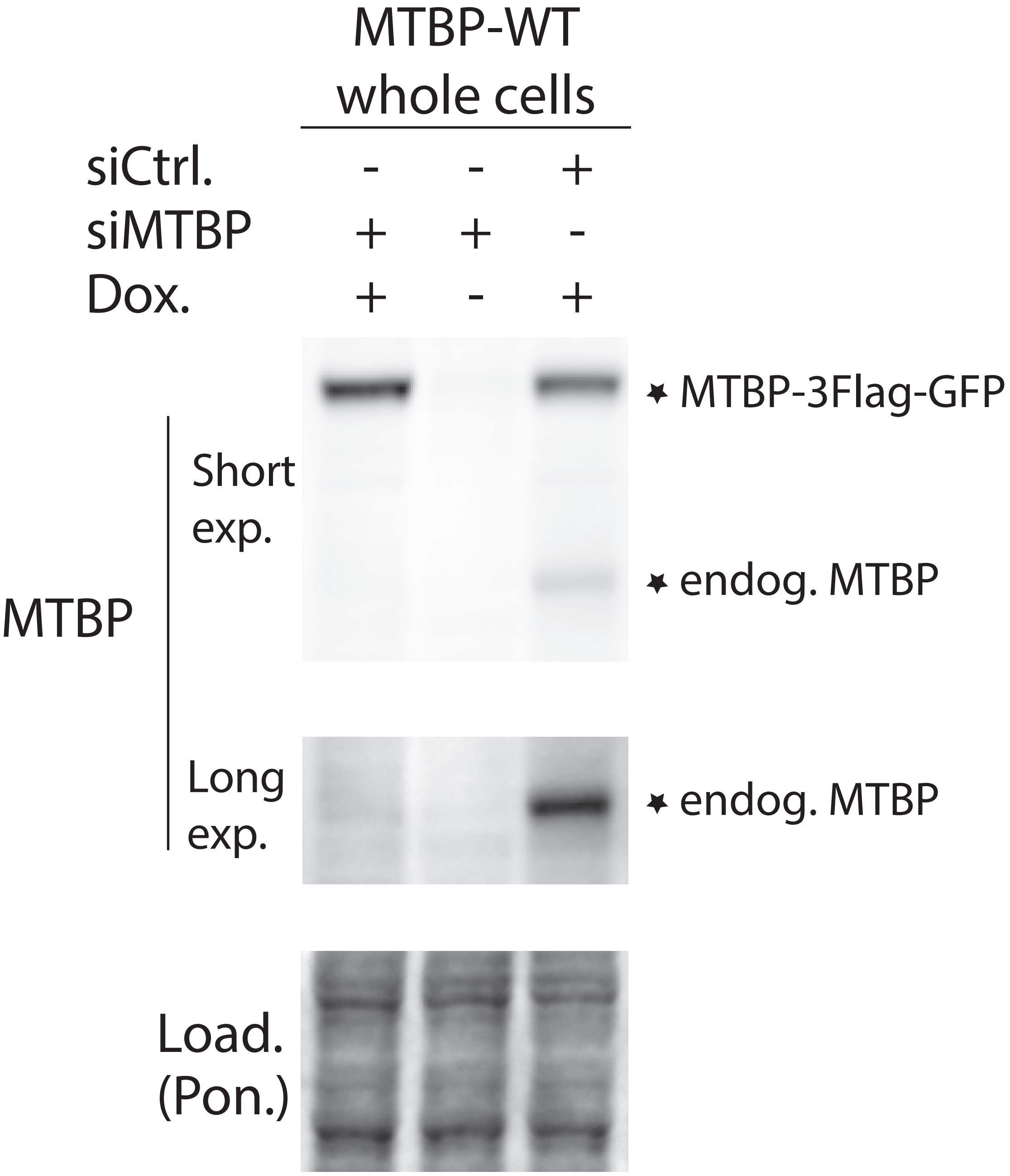
MTBP-WT
whole cells
siCtrl. - - +
siMTBP + + -
Dox. + - +
MTBP-3Flag-GFP
Short exp.
endog. MTBP
MTBP
Long exp.
endog. MTBP
Load. (Pon.)

Fig. S4: Expression levels of checkpoint kinase site MTBP mutants are similar to MTBP-WT. Cell lysates of Hela Flip-In T-Rex cell expressing MTBP-wild type (WT), MTBP-14D, MTBP-10D, MTBP-4D, MTBP-1D and MTBP-3D were analysed by immunoblotting using anti-MTBP (12H7) and Ponceau (Pon.) staining.

Figure S4

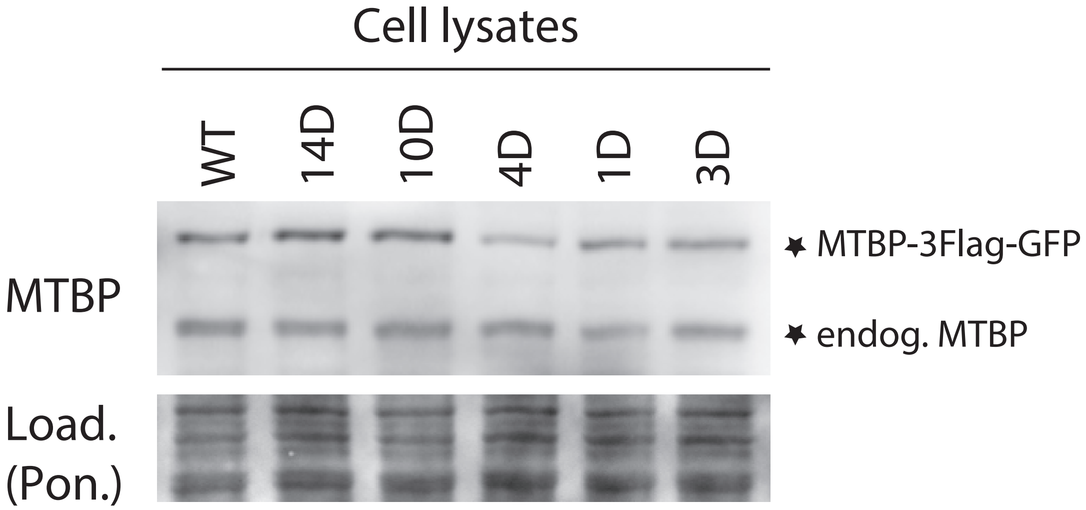
Cell lysates
WT
14D
10D
4D
1D
3D
MTBP
MTBP-3Flag-GFP
endog. MTBP
Load.
(Pon.)

Fig. S5: ATR inhibitor VE-821 does not restore DNA synthesis in MTBP-14D cells

A) Individual PI-BrdU density flow cytometry plots quantified for Fig. 3C. Upon siMTBP treatment, MTBP-WT and MTBP-14A cells showed normal profiles whilst parental control cells and MTBP-14D cells showed heavily compromised BrdU incorporation, regardless of presence of the ATR inhibitor VE-821 (6 µM) for 2 h.

B) Whole cell lysates of cells treated as in A were immunoblotted to determine ATR activity levels by anti-phospho-S345-Chk1 antibody. Treatment with 2 mM hydroxyurea (HU) served as a control that induces strong ATR activity. Parental cells and MTBP-14D cells, but not MTBP-WT and 14A cells, induced detectable ATR activity, albeit less strong than HU. This suggests that severely compromising origin firing leads to ATR-checkpoint activation independently on the presence of MTBP-14D. Adding 6 µM VE-821 decreased ATR activity below levels detected in siCtr-treated MTBP-WT cells. This suggests that a) VE-821 inhibits ATR, and b) normally growing Hela cells have basal ATR-checkpoint activity.

Figure S5

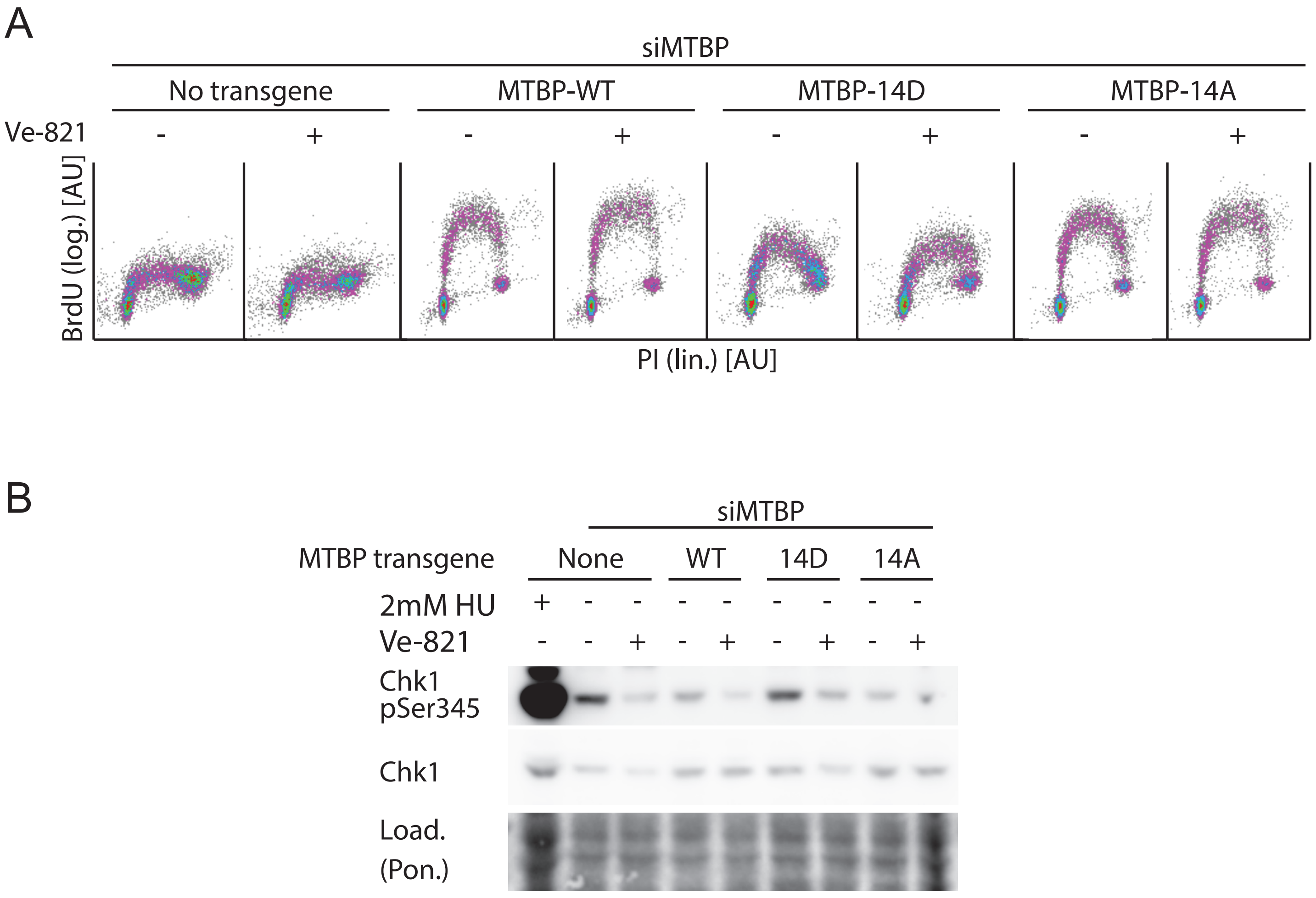
A
siMTBP
No transgene
MTBP-WT
MTBP-14D
MTBP-14A
Ve-821
-
+
-
+
-
+
-
+
BrdU (log.) [AU]
PI (lin.) [AU]
B
siMTBP
MTBP transgene
None
WT
14D
14A
2mM HU
+ - - - - - - - -
Ve-821
- - + - + - + - +
Chk1 pSer345
Chk1
Load. (Pon.)

Fig. S6: Forced MTBP dimerization rescues phospho-mimetic MTBP checkpoint site mutants

A) Expression levels of mutant and WT MTBP-GST and GFP are similar. GST-tagged MTBPs are always lower expressed than GFP-tagged MTBPs. Whole cell lysates of Hela Flip-In T-Rex cell expressing MTBP-wild type (WT), MTBP-ΔC150, MTBP-5m, MTBP-4A, MTBP-4D, MTBP-14A, or MTBP-14D were analysed by immunoblotting using anti-MTBP (12H7) and Ponceau (Pon.) staining.

B/C) Forced MTBP dimerization of MTBP by GST fusion rescues deletion of the MTBP C-terminus (ΔC150) and checkpoint kinase site aspartate mutants (4D, 14D), but not a Treslin non-binding MTBP mutant (5m). Quantification of replication by BrdU-PI-flow cytometry (B) or cell cycle distribution (C) of cell lines described in A. Most important samples are also shown in Figure 3. Replication activity and cell cycle distribution of Hela Flip-In T-Rex cells was measured after replacement of endogenous MTBP with indicated mutants. Cells were treated with control siRNA (siCtr) or siRNA against MTBP (siMTBP) and doxycycline, stained with anti-5 bromo-2'deoxyuridine (BrdU) after pulse-labelling and by propidium iodide (PI). Error bar: SEM; n= 4 (no transgene); 4 (MTBP-WT); 4 (MTBP-ΔC150); 4 (MTBP-5m), 4 (MTBP-4A-GS4-GFP); 3 (MTBP-4A-GS4-GST); 3 (MTBP-4D-GS4-GFP); 5 (MTBP-4D-GS4-GST); 3 (MTBP-14A), 3 (MTBP-14D-GS4-GFP), 5 (MTBP-14D-GS4-GST). Significance tests as in 2D/E. In (C) are indicated differences in G2/M population distribution.

Figure S6

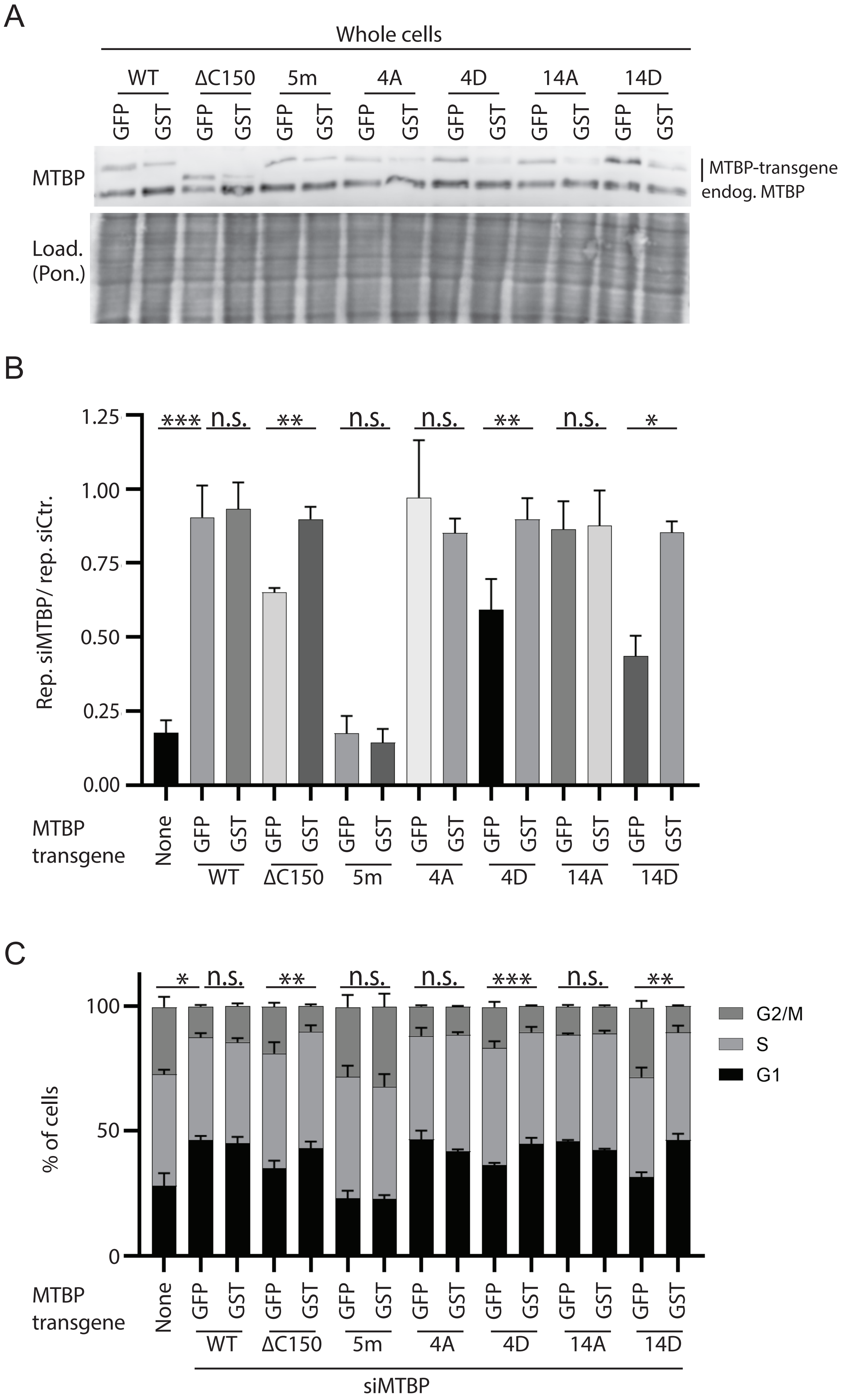
A
Whole cells
WT
ΔC150
5m
4A
4D
14A
14D
GFP
GST
MTBP
MTBP-transgene
endog. MTBP
Load. (Pon.)
B
Rep. siMTBP/ rep. siCtr.
1.25
1.00
0.75
0.50
0.25
0.00
*** n.s. ** n.s. n.s. ** n.s. *
MTBP transgene
None
C
% of cells
100
50
0
* n.s. ** n.s. n.s. *** n.s. **
G2/M
S
G1
siMTBP

Fig S7: Measuring radio-resistant DNA synthesis by BrdU-flow cytometry

A/B) Reduction of replication by ionising irradiation is dose-dependent. BrdU-PI-flow cytometry density plots of HeLa Flip-In T-Rex cells radiated at 130 kV, with a 0,5 mm-aluminium filter at a dose rate of 1 Gy/72 sec (RX-650 Faxitron), with a total dose of 0, 6 , 10 or 20 Gy, and BrdU pulse labelled 1h after irradiation, stained with anti-5 bromo-2'deoxyuridine (BrdU) by propidium iodide (PI). B) shows a decrease in BrdU signals with increasing IR doses. Relative quantification of BrdU signals used normalisation to cells treated with 0 Gy. Log., logarithmic scale; lin., linear scale; [AU], arbitrary units.

C/D) Reduction of replication by ionising radiation takes effect at 30 minutes after irradiation, is maximal around 3 h and recovers at later time points. Quantification of replication activity by BrdU-PI-flow cytometry as described in A of HeLa Flip-In T-Rex cells irradiated at 130 kV, with a 3 mm-aluminium filter at a dose rate of 1 Gy/50 sec (Philips) with a total dose of 6 Gy, and BrdU pulse labelled 0,5 h, 1 h and 3 h (C), or 3 h, 5 h, and 7 h (D) after irradiation, and stained as in A. Because a different x-ray unit with a different aluminium filter was used, 6 Gy here are equivalent to 20 Gy in A/B.

E) Reduction of replication by ionising radiation is dependent on the ATR kinase. Treatment of irradiated cells with ATR inhibitor VE-821 (6 µM) rescued replication activity to same levels as non-irradiated cells 1h after IR treatment. Flow cytometry PI-BrdU density plots of HeLa Flip-In T-Rex cells irradiated at 130 kV, with a 0,5 mm-aluminium filter at a dose rate of 1 Gy/72 sec (RX-650 Faxitron), with a total dose of 0 or 20 Gy, treated with DMSO or ATR inhibitor VE-821, and stained as in A. Quantification of BrdU signals are normalisations to cells treated with 0 Gy and DMSO.

Figure S7

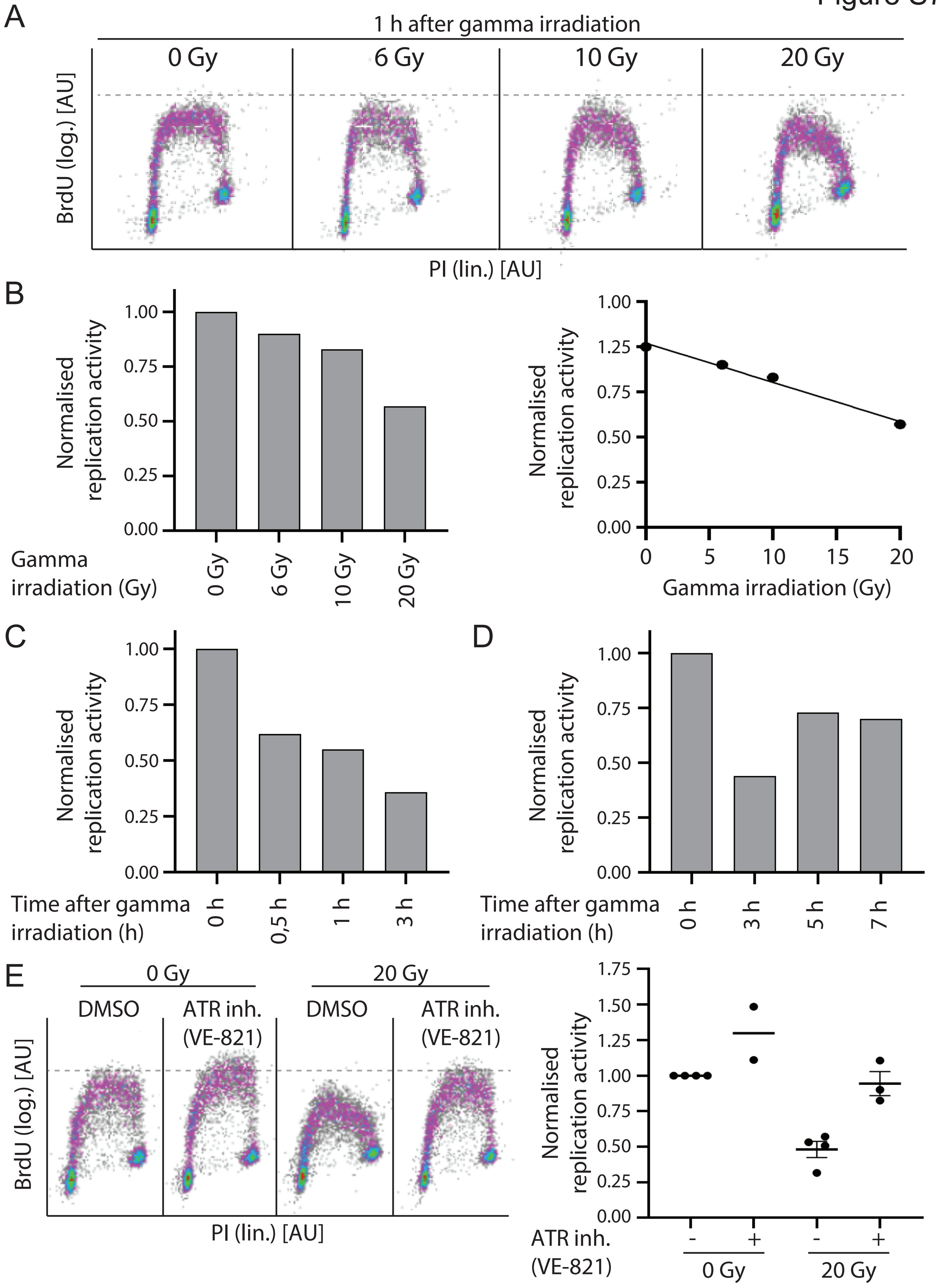
A
1 h after gamma irradiation
0 Gy
6 Gy
10 Gy
20 Gy
BrdU (log.) [AU]
PI (lin.) [AU]
B
Normalised replication activity
1.00
0.75
0.50
0.25
0.00
Gamma irradiation (Gy)
0 Gy
6 Gy
10 Gy
20 Gy
Normalised replication activity
1.00
1.25
0.75
0.50
0.25
0.00
0
5
10
15
20
Gamma irradiation (Gy)
C
Normalised replication activity
1.00
0.75
0.50
0.25
0.00
Time after gamma irradiation (h)
0 h
0,5 h
1 h
3 h
D
Normalised replication activity
1.00
0.75
0.50
0.25
0.00
Time after gamma irradiation (h)
0 h
3 h
5 h
7 h
E
0 Gy
20 Gy
DMSO
ATR inh. (VE-821)
DMSO
ATR inh. (VE-821)
BrdU (log.) [AU]
PI (lin.) [AU]
Normalised replication activity
1.75
1.50
1.25
1.00
0.75
0.50
0.25
0.00
ATR inh. (VE-821)
-
+
-
+
0 Gy
20 Gy

Fig. S8: Characterising ATR signalling and replication fork speed in MTBP-14A, 6A and 6D mutant cell lines

A) MTBP-14A cells show normal basic levels and normal response dynamics of ATR-checkpoint activation upon hydroxyurea (HU) treatment. Whole cell lysates of cells treated with buffer or indicated concentrations of hydroxyurea were immunoblotted using anti-Chk1 and anti-pS345-Chk1 antibodies. Ponceau staining served as a loading control. HU induced detectable Chk1 phosphorylation from 100 µM in both cell lines.

B) MTBP-6D and 6A cells have normal basic levels and normal response dynamics of ATR-checkpoint activation upon hydroxyurea (HU) treatment. MTBP-6D, 6A and WT cells were treated and analysed as described in A. No difference between WT and the mutants could be observed.

C) Replication fork speeds in different MTBP phospho-mutant cell lines. Average speed of replication forks in the phospho-site mutants and Cdk8 non-binding mutant is similar to MTBP-WT. Quantification of average fork speed (kbp/min) by DNA combing with siMTBP-treated Hela Flip-In T-Rex cells expressing siMTBP resistant C-terminally 3xFlag-TEV2-GFP-tagged MTBP-wild type (WT), MTBP-14A, MTBP-cdk8bm, MTBP-6D, or MTBP-6A. Error bars: SEM; n = 3; Significance tests as in 2D/E.

Figure S8

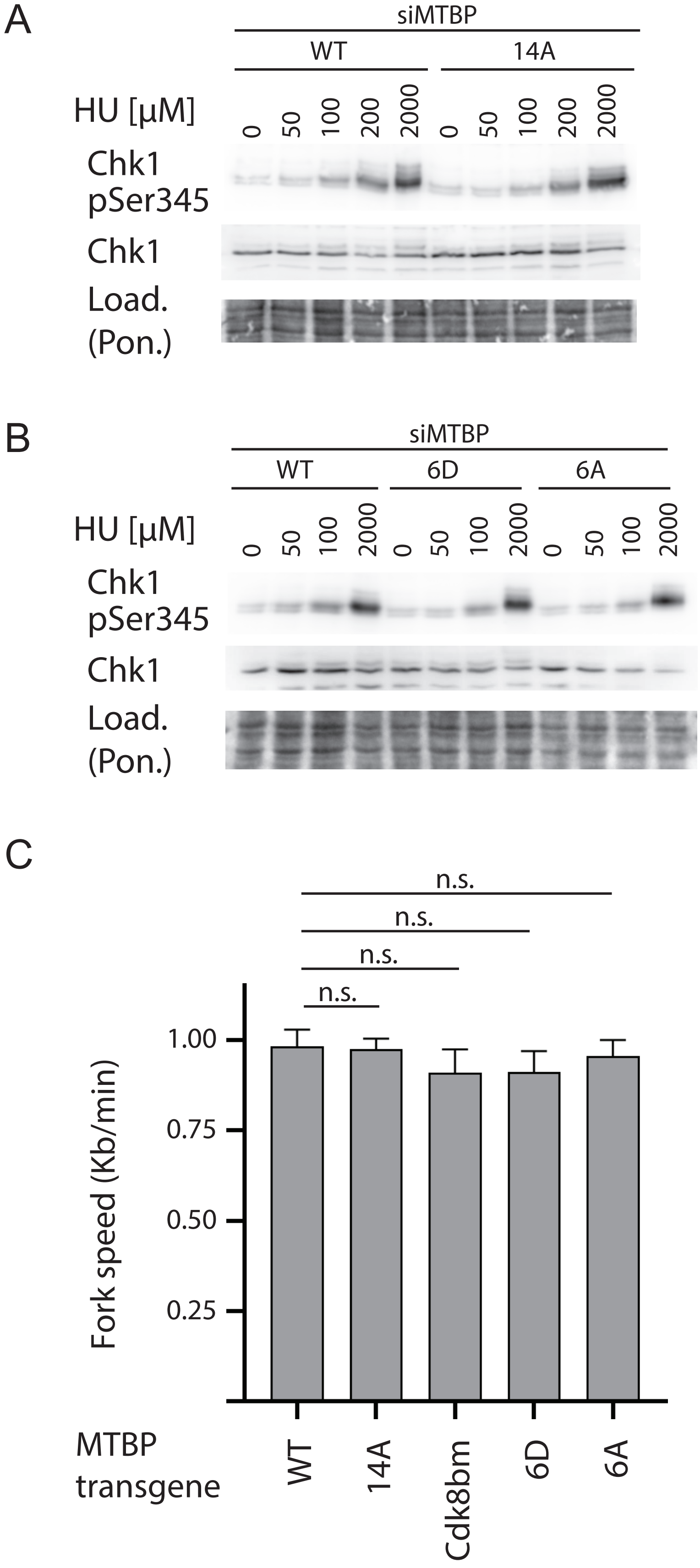
A
siMTBP
WT
14A
HU [µM]
0
50
100
200
2000
0
50
100
200
2000
Chk1 pSer345
Chk1
Load. (Pon.)
B
siMTBP
WT
6D
6A
HU [µM]
0
50
100
2000
0
50
100
2000
0
50
100
2000
Chk1 pSer345
Chk1
Load. (Pon.)
C
n.s.
n.s.
n.s.
n.s.
1.00
0.75
0.50
0.25
Fork speed (Kb/min)
MTBP transgene
WT
14A
Cdk8bm
6D
6A

Fig. S9: MTBP is phosphorylated in cell lysates

A) Phosphorylation-mediated gel shifts of MTBP and Treslin in mitotic cells depend on M-CDK. Data shown in Fig 5A. For better visualisation of the gel shift, a dashed line was introduced indicating the running behaviour of phospho-MTBP. To reveal phospho-shifts SDS PAGE using 3-8% Tris-acetate Criterion gels (Biorad) was used, and the degree of shift varied between gels. Cells arrested in mitosis with nocodazole (Noc) or asynchronous cells were treated for 30 minutes with DMSO, low (9μM) or high concentrations (90μM) of RO-3306 (RO.), roscovitine (Rosc.) or senexin A (Sen.). After cell lysis lysates were treated with lambda phosphatase (PPase) where indicated before immunoblotting using antibodies against MTBP (12H7) and Treslin (148). MTBP and Treslin from cells treated with lambda phosphatase or Cdk1 inhibitors (RO-3306 or roscovitine) showed fastest gel running behaviour than MTBP from cells treated with DMSO or Cdk8 inhibitor senexin A, indicating that both proteins are phosphorylated by Cdk1 in mitosis. Ponceau staining shows that stained unspecific bands run at the same speed in all lanes.

B) Endogenous MTBP showed a phosphorylation-mediated gel shifts in S-phase. Cells were arrested with a nocodazole-thymidine block before release into S-phase for 4h before lysis, lambda phosphatase treatment and addition of Laemmli sample buffer. For better visualisation of gel shifts dashed lines were introduced that indicate running behaviour of phospho-MTBP. (i) MTBP from cells treated with phosphatase showed slightly faster gel modility than cells treated with DMSO. The phosphorylation-mediated gel shifts of MTBP and Treslin in S phase cells is smaller than in mitotic cells (A), which precluded conclusions about inhibitor effects.

(ii) Second experiment confirming (i). Here, S phase cell lysates treated with DMSO or phosphatase are shown. Here, indicated cells were treated with the phosphatase inhibitors tautomycin (tauto.) and okadaic acid (ok.) before lysis in the hope to increase the phospho-shift, which was ineffective.

C) Cells used in B were stained with anti-5 bromo-2'deoxyuridine (BrdU) after pulse-labelling. Quantification of BrdU intensity by flow-cytometry showed that 71% of the cells released from thymidine for 4h had started replication in comparison to 5% of cells that remained arrested in thymidine. This indicates that the majority of the cells used in B was in S-phase.

Figure S9

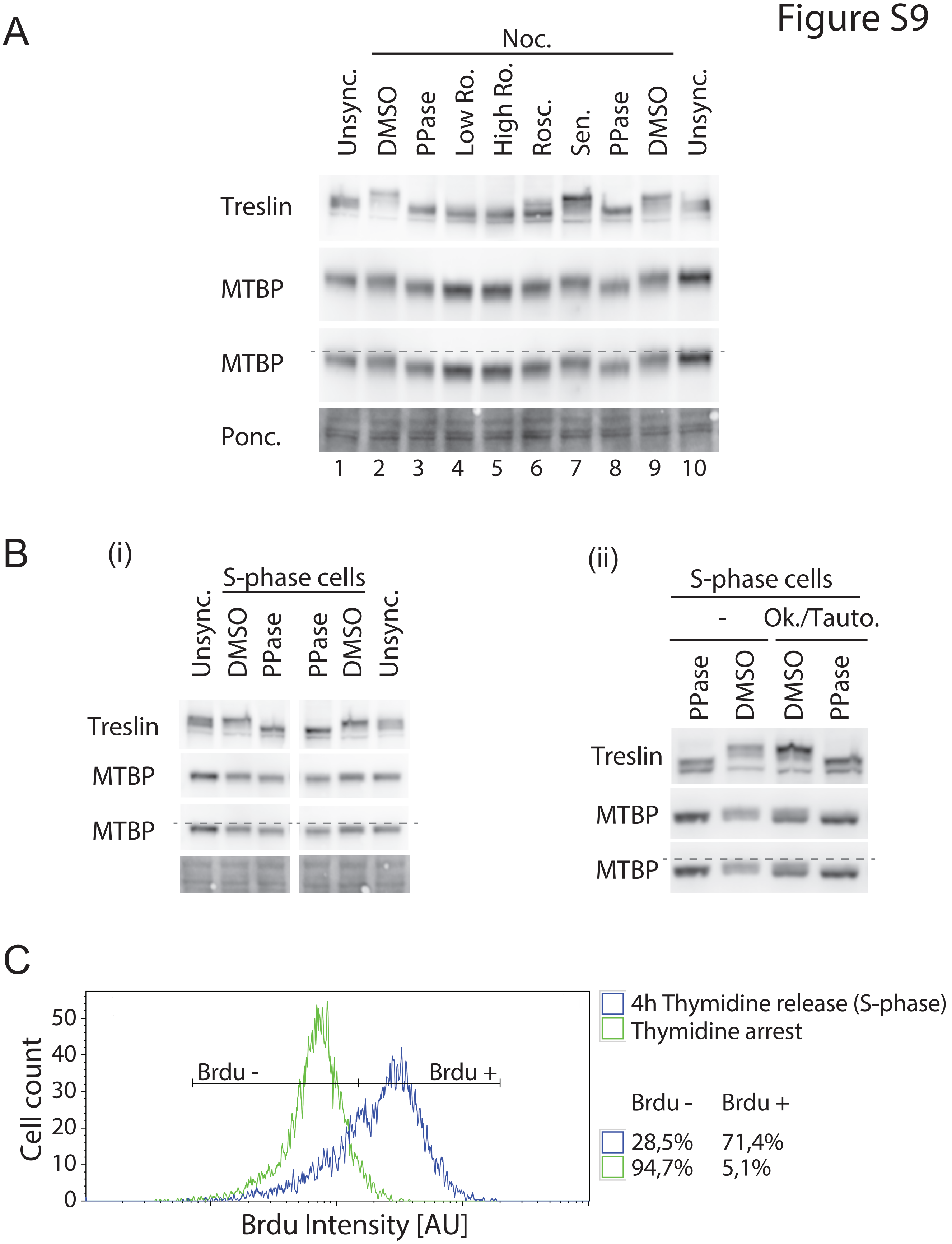
A
Noc.
Unsync.
DMSO
PPase
Low Ro.
High Ro.
Rosc.
Sen.
PPase
DMSO
Unsync.
Treslin
MTBP
MTBP
Ponc.
1 2 3 4 5 6 7 8 9 10
B
(i)
S-phase cells
Unsync.
DMSO
PPase
PPase
DMSO
Unsync.
Treslin
MTBP
MTBP
(ii)
S-phase cells
-
Ok./Tauto.
PPase
DMSO
DMSO
PPase
Treslin
MTBP
MTBP
C
Cell count
0 10 20 30 40 50
Brdu -
Brdu +
Brdu Intensity [AU]
4h Thymidine release (S-phase)
Thymidine arrest
Brdu - Brdu +
28,5% 71,4%
94,7% 5,1%

Fig. S10: Scatter plots of inter-origin distance measurements by DNA combing in different MTBP phospho-mutant cell lines

In 4C and 6D few data points were cropped out for better visibility. Here, the uncropped images are shown. Scatter plots of individual IOD measurement samples are shown. (A) quantification in Fig. 4 C(ii); (B) quantification in Fig. 6F(ii).

Figure S10

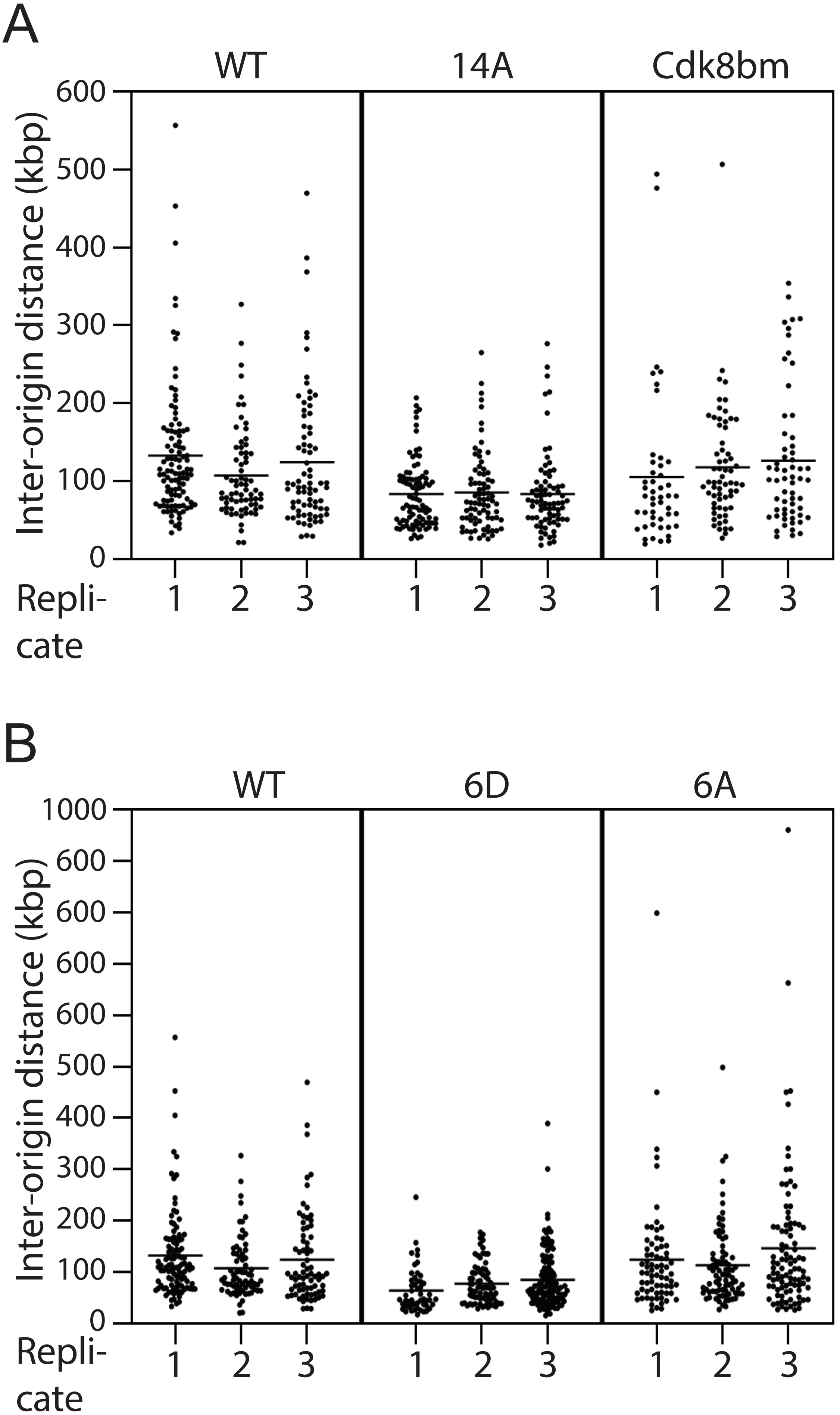

A
WT
14A
Cdk8bm
Inter-origin distance (kbp)
600
500
400
300
200
100
0
Repli-
cate
1
2
3
B
WT
6D
6A
Inter-origin distance (kbp)
1000
600
600
600
600
500
400
300
200
100
0
Repli-
cate
1
2
3

Fig. S11: Uncropped images of the immunoblots and radiograms presented in the main and supplementary figures.

Figure S11

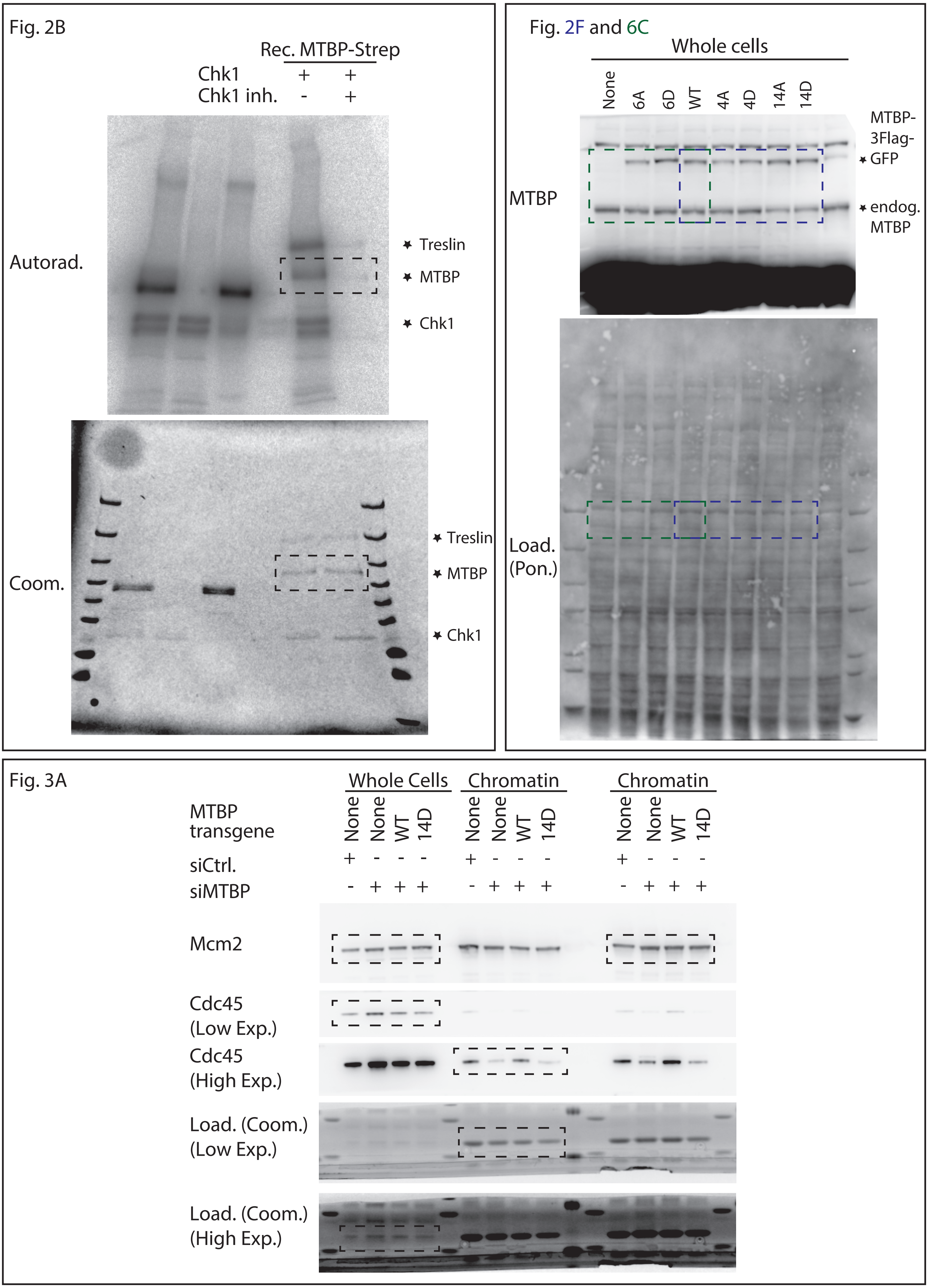

Fig. 2B
Rec. MTBP-Strep
Chk1 + +
Chk1 inh. - +
Autorad.
Treslin
MTBP
Chk1
Coom.
Treslin
MTBP
Chk1
Fig. 2F and 6C
Whole cells
None 6A 6D WT 4A 4D 14A 14D
MTBP
MTBP-3Flag-GFP
endog. MTBP
Load. (Pon.)
Fig. 3A
Whole Cells
Chromatin
Chromatin
MTBP transgene
None None WT 14D
siCtrl.
siMTBP
+ - - -
- + + +
Mcm2
Cdc45 (Low Exp.)
Cdc45 (High Exp.)
Load. (Coom.) (Low Exp.)
Load. (Coom.) (High Exp.)

Fig. 3B

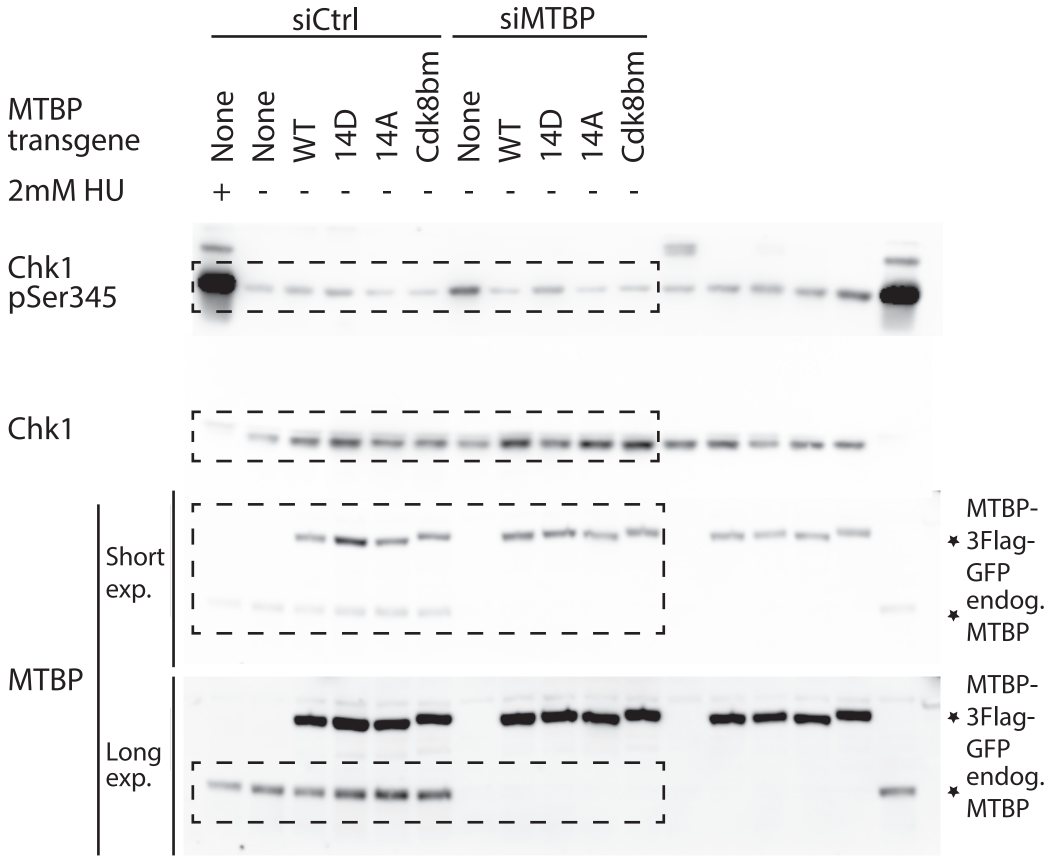
siCtrl
siMTBP
MTBP transgene
None
None
WT
14D
14A
Cdk8bm
None
WT
14D
14A
Cdk8bm
2mM HU
+
-
Chk1 pSer345
Chk1
Short exp.
Long exp.
MTBP
MTBP-3Flag-GFP
endog. MTBP

Fig. 3D

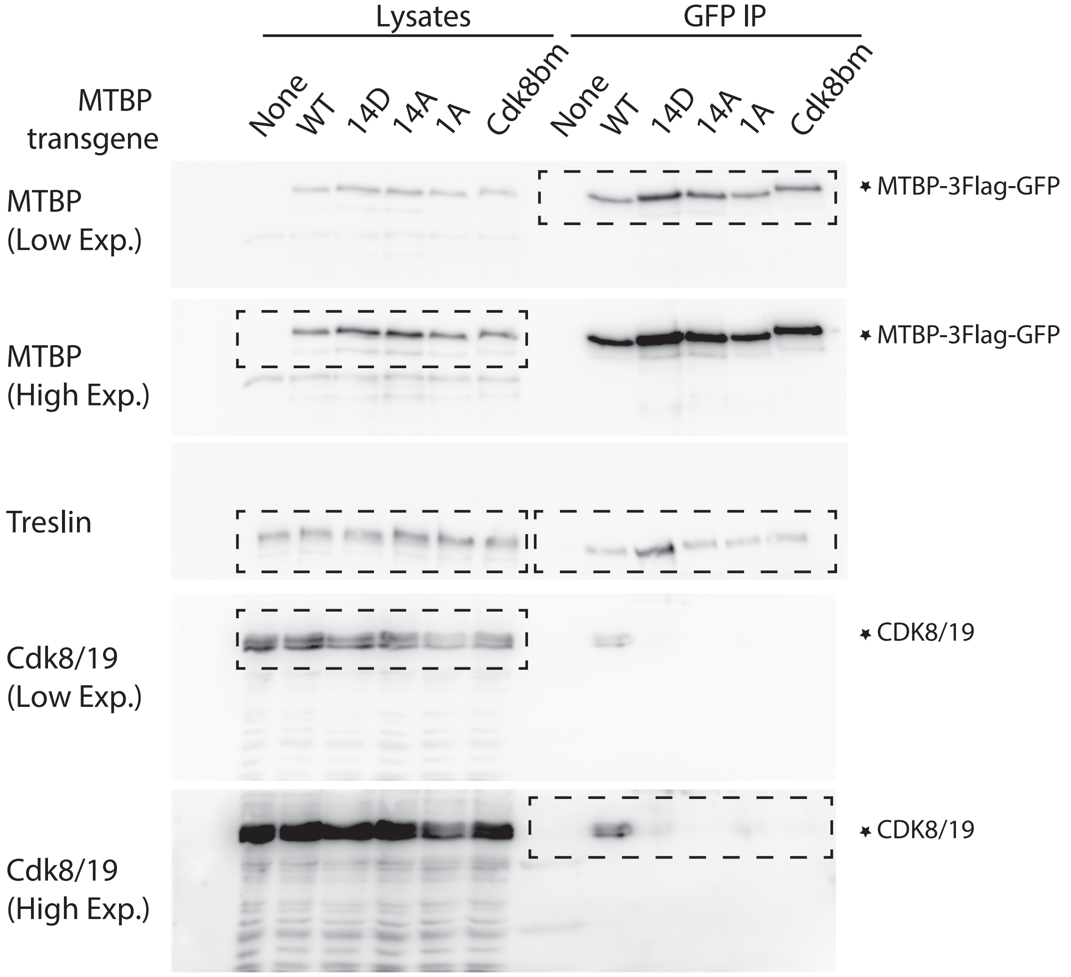
Lysates
GFP IP
MTBP transgene
None
WT
14D
14A
1A
Cdk8bm
MTBP (Low Exp.)
MTBP (High Exp.)
Treslin
Cdk8/19 (Low Exp.)
Cdk8/19 (High Exp.)
MTBP-3Flag-GFP
CDK8/19

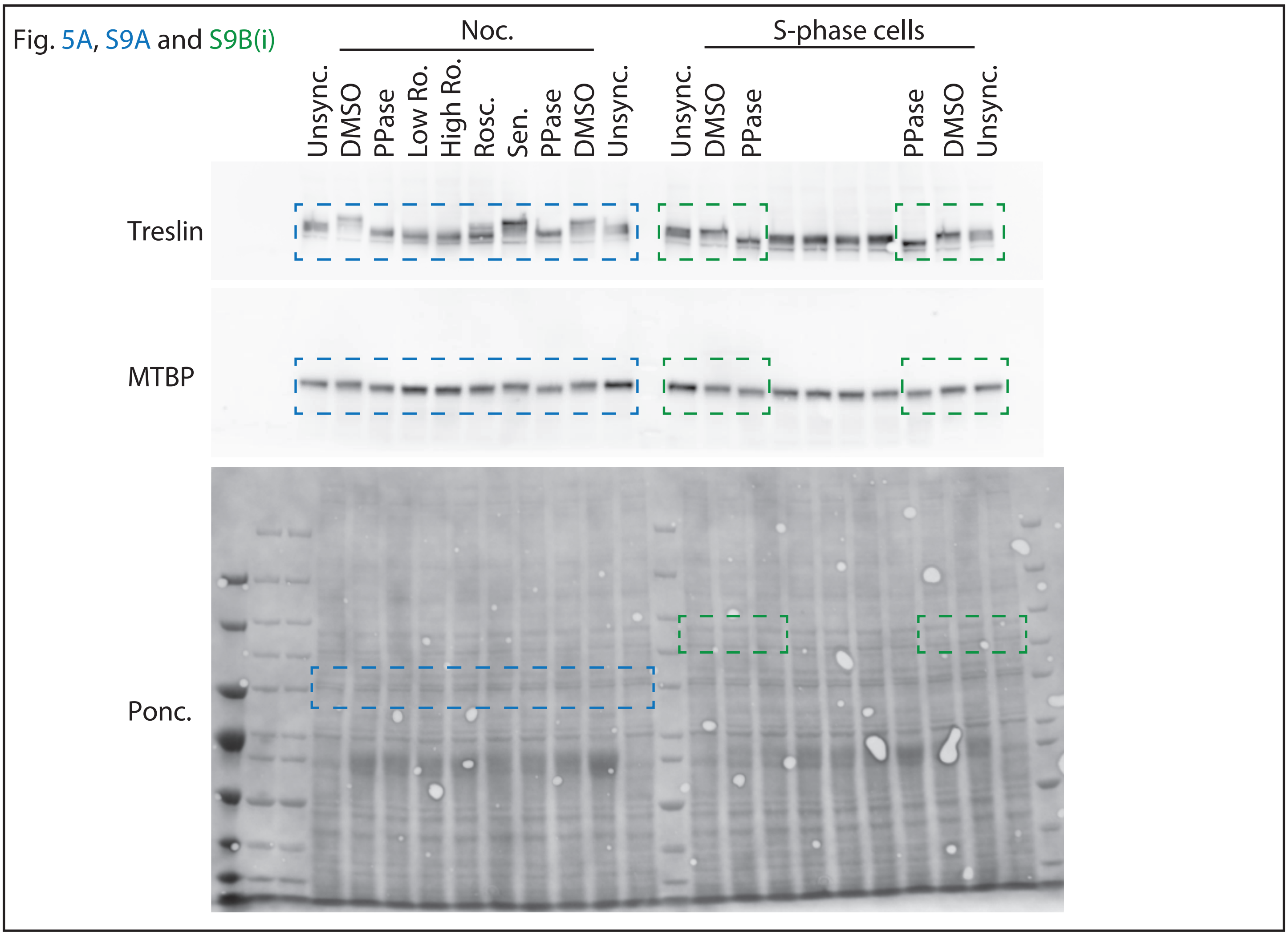
Fig. 5A, S9A and S9B(i)
Noc.
S-phase cells
Unsync. DMSO PPase Low Ro. High Ro. Rosc. Sen. PPase DMSO Unsync.
Unsync. DMSO PPase PPase DMSO Unsync.
Treslin
MTBP
Ponc.

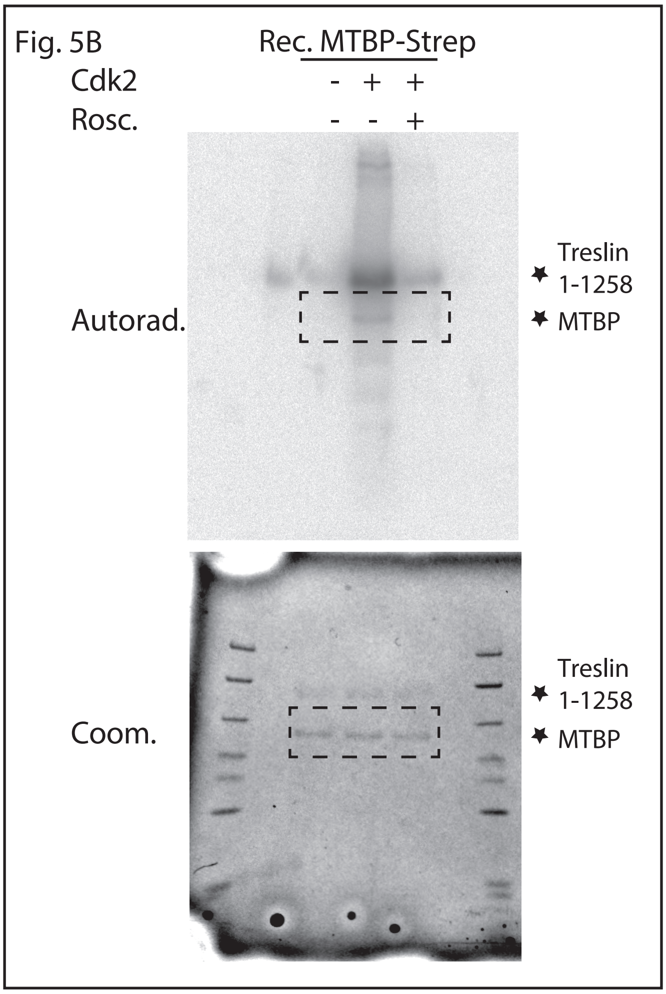
Fig. 5B
Rec. MTBP-Strep
Cdk2 - + +
Rosc. - - +
Autorad.
Treslin 1-1258
MTBP
Coom.
Treslin 1-1258
MTBP

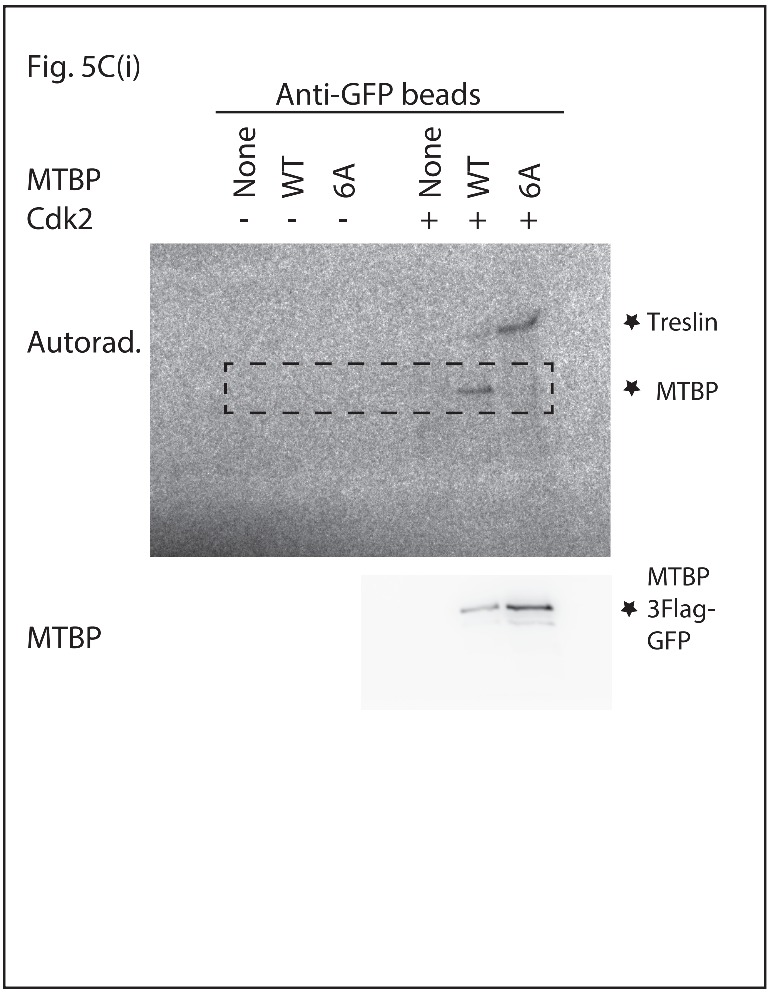
Fig. 5C(i)
Anti-GFP beads
MTBP None WT 6A None WT 6A
Cdk2 - - - + + +
Autorad.
Treslin
MTBP
MTBP
MTBP 3Flag-GFP

Fig. 5D

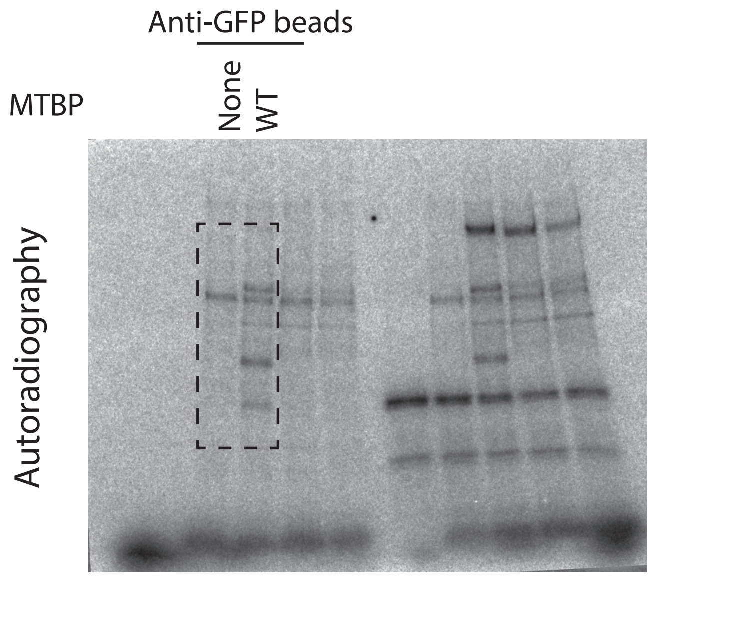

Fig. 5E and 5F

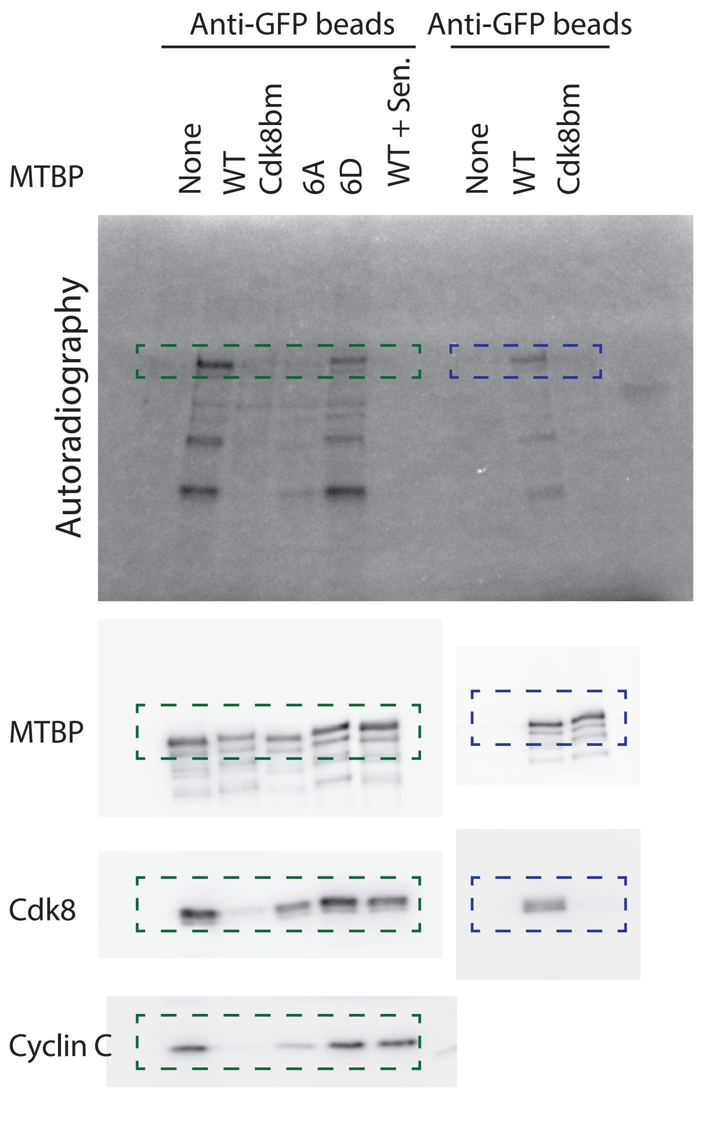

Fig. S1C

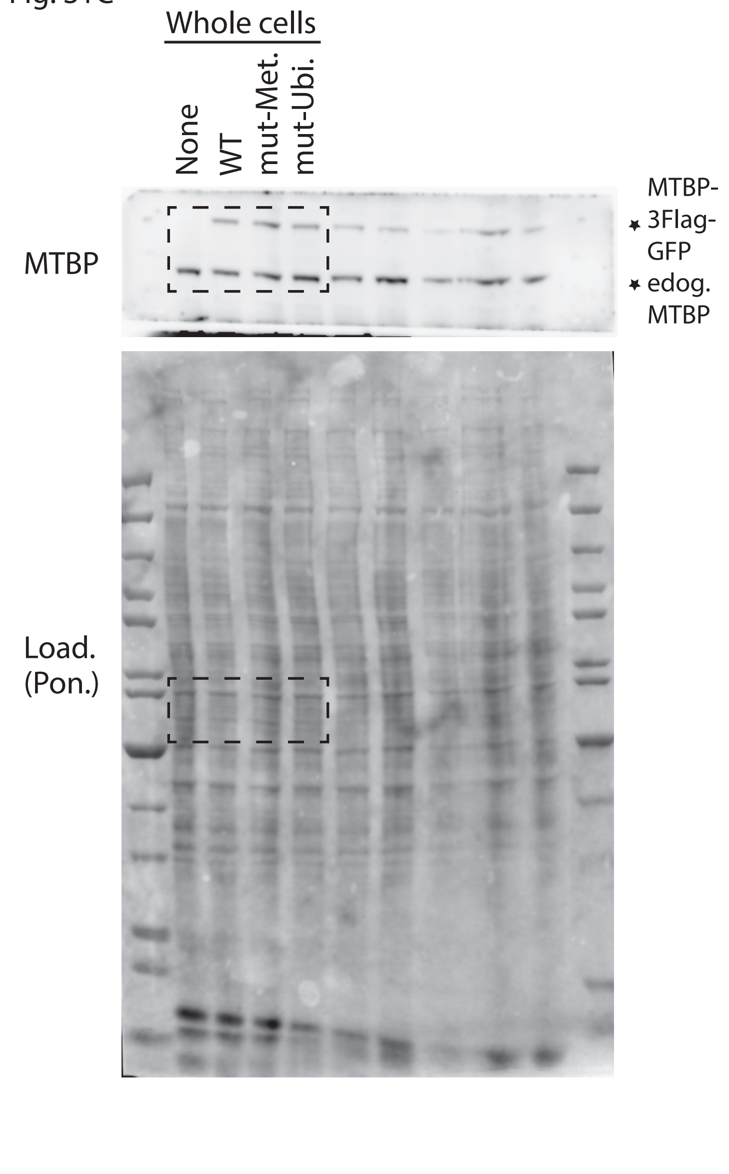

Fig. S2

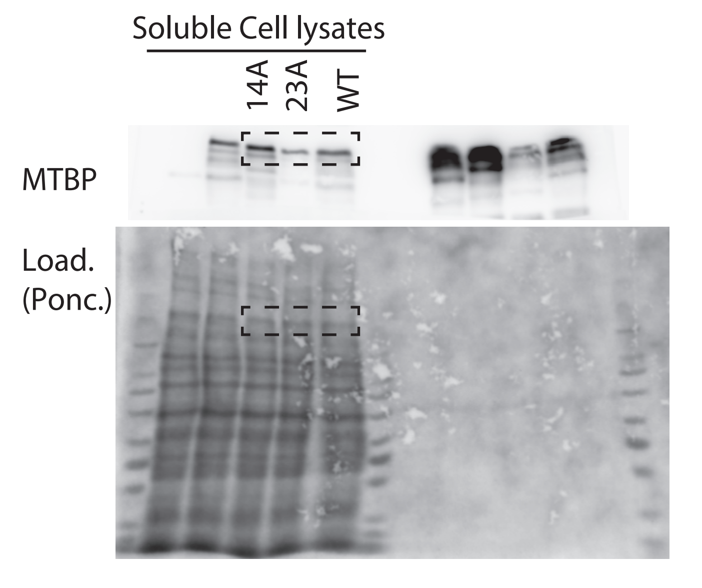

Fig. S3

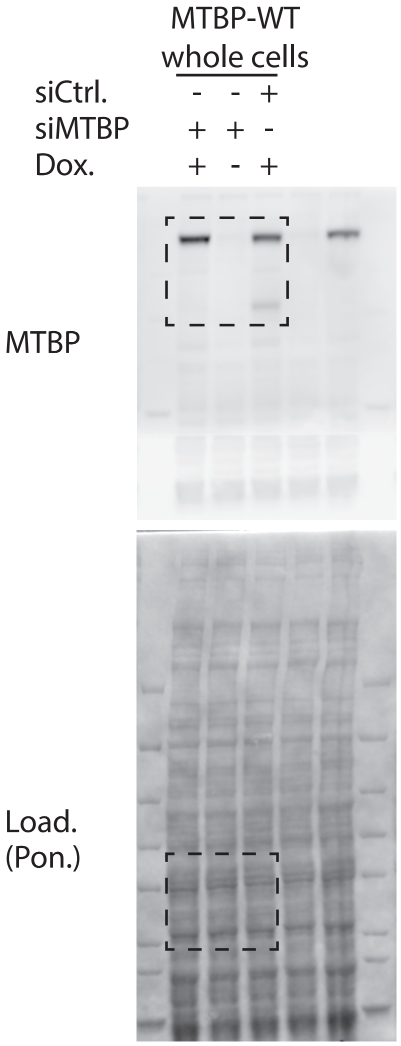

Fig. S4

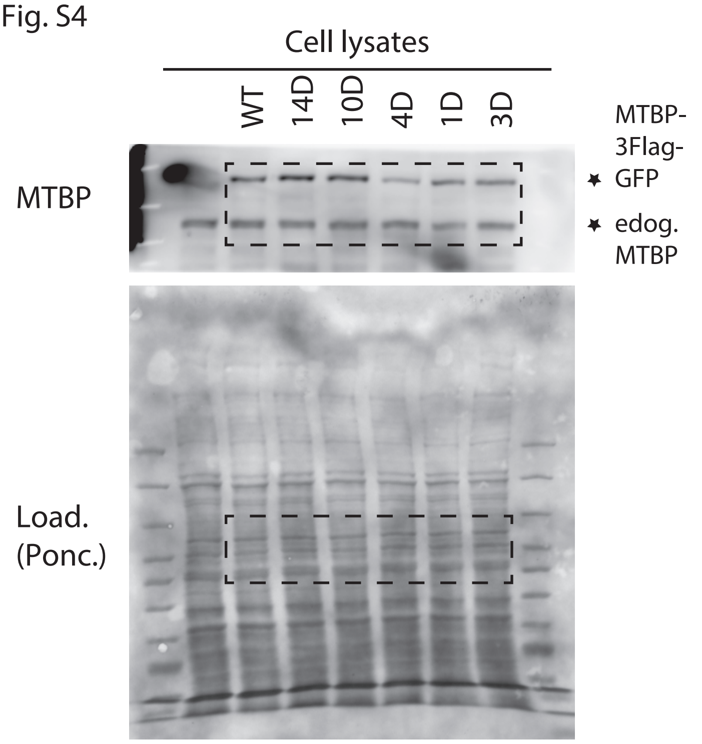

Fig. S5B

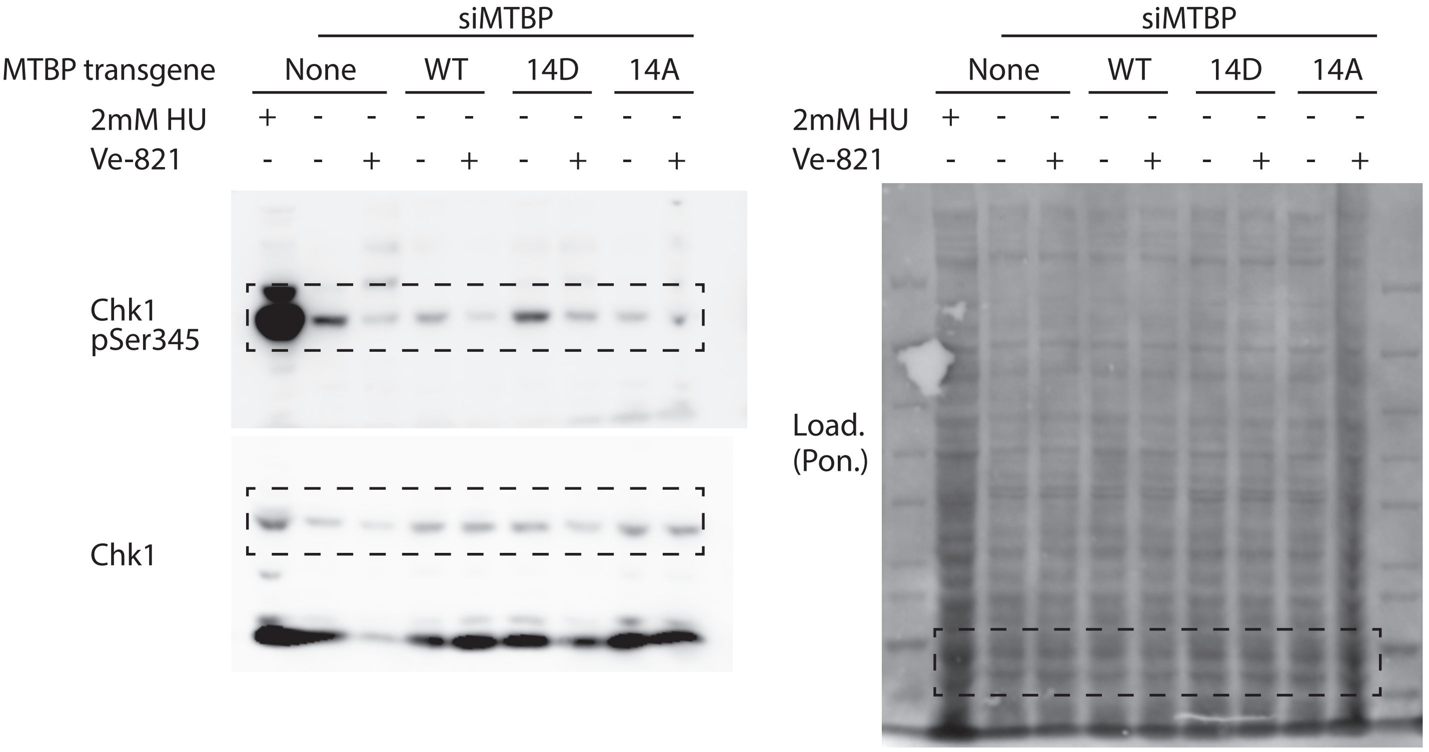

Fig. S6A

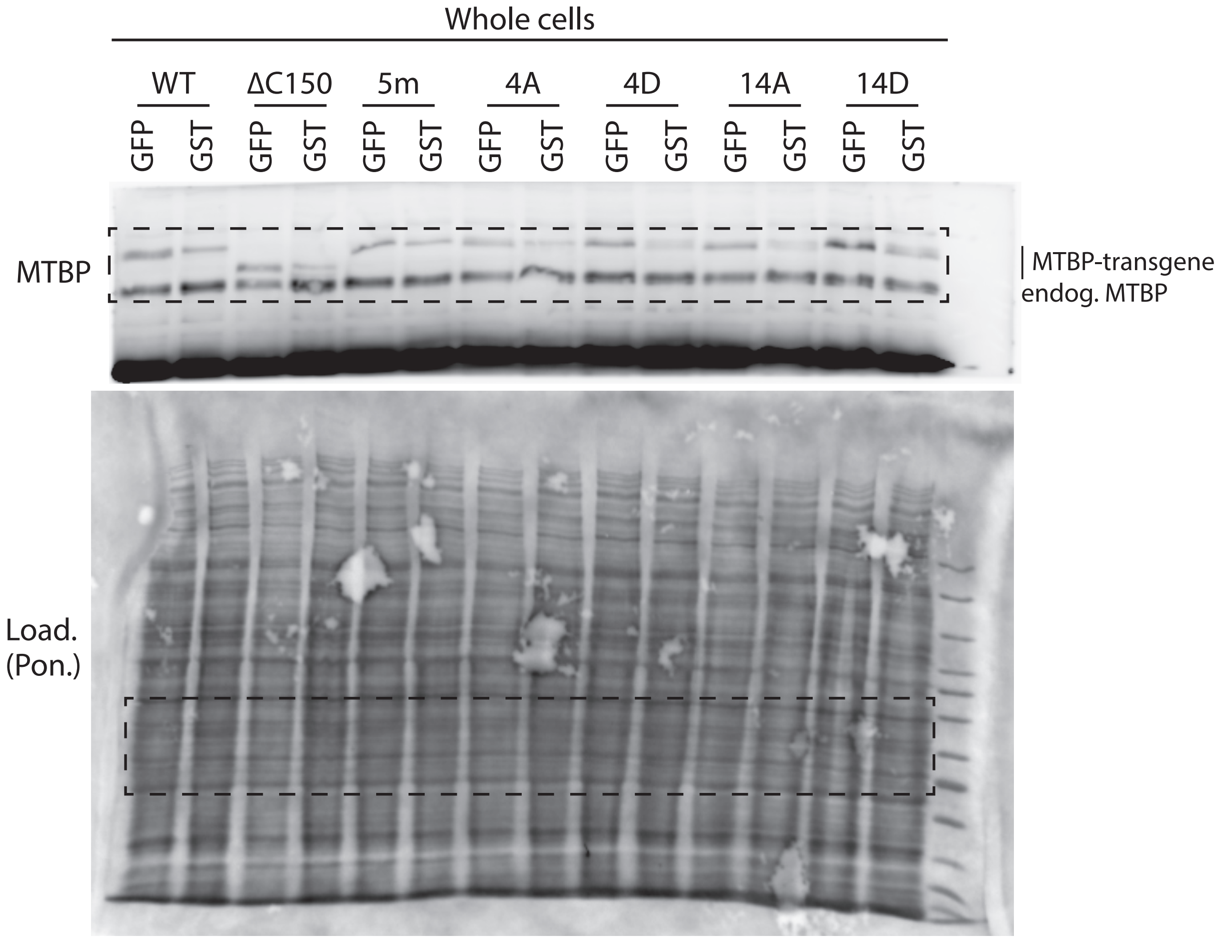

Fig. S8A

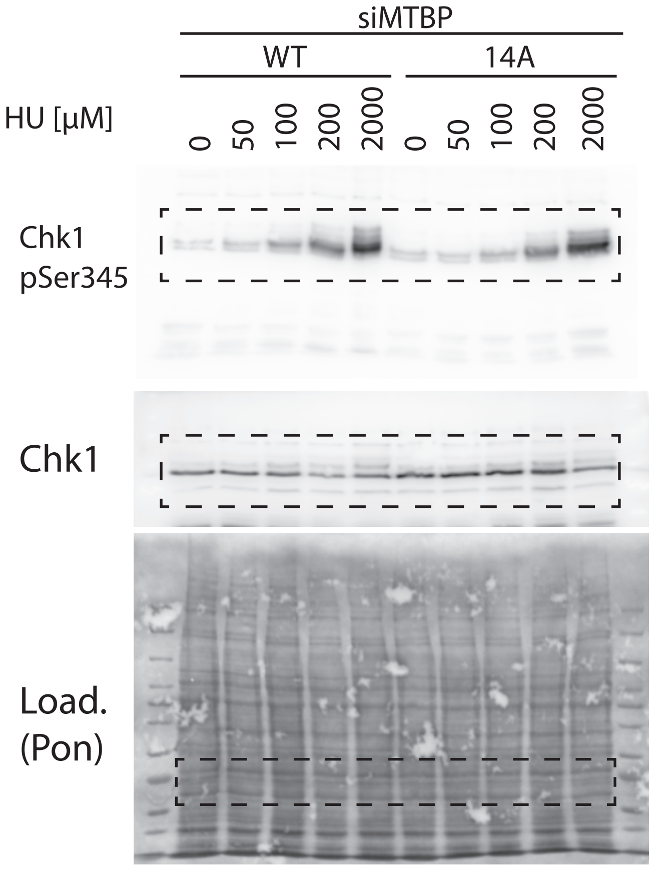

Fig. S8B

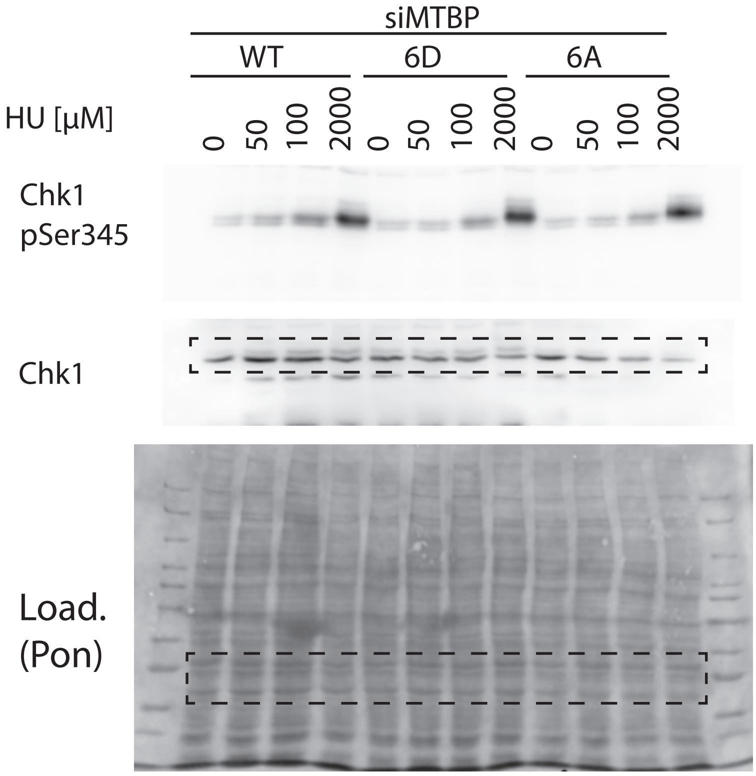

Supplement: Supplementary file 1 — Supplementary Information. [file 41598_2021_83287_MOESM1_ESM.pdf]
